# Coordination-Driven Assembly of Alginate Networks: From Egg-Box Structures to Bioactive Delivery Applications

**DOI:** 10.3390/gels12090774

**Published:** 2026-08-28

**Authors:** İrem Toprakçı, Ebru Kurtulbaş, Rabia Nur Bozkurt, Selin Şahin

**Affiliations:** 1 Department of Chemical Engineering, Faculty of Engineering, Istanbul University-Cerrahpaşa, Avcılar, 34320 Istanbul, Türkiye; irem.toprakciyuksel@iuc.edu.tr (İ.T.); ebru.kurtulbas@iuc.edu.tr (E.K.); rabia.bozkurt@istun.edu.tr (R.N.B.); 2 Department of Chemical Engineering, Faculty of Engineering and Natural Sciences, Istanbul Health and Technology University, Beyoğlu, 34440 Istanbul, Türkiye

**Keywords:** alginate, Ca^2+^-mediated crosslinking, egg-box model, microparticles, bioactive delivery

## Abstract

Alginate is a biodegradable and renewable natural polysaccharide that has been extensively investigated for the design of macromolecular delivery systems. This review presents an overview of alginate-based microparticles regarding the relationship between molecular structure, gelation behavior, and functional performance. The impacts of main structural parameters (mannuronic to guluronic acid (M/G) ratio, molecular weight, and block distribution) are discussed comprehensively. Particular attention is given to Ca^2+^-mediated ionic gelation, including egg-box junction zone formation and the development of three-dimensional hydrogel networks. Different production strategies such as external and internal gelation, emulsification, and microfluidic approaches are evaluated in terms of their impact on particle morphology and network homogeneity. In addition, the effects of formulation and process parameters on encapsulation efficiency, mechanical stability, and mass transfer behavior are analyzed. Furthermore, release mechanisms are discussed in relation to network structure and polymer-solute interactions. The environmental significance of alginate-based systems is also emphasized as sustainable alternatives to synthetic polymeric carriers.

## 1. Introduction

Bioactive compounds (polyphenols, carotenoids, essential oils, vitamins, peptides, probiotics, and various pharmaceutical agents) have attracted considerable attention because of their nutritional, functional, and therapeutic potential. However, many of these compounds are sensitive to environmental and processing conditions. Therefore, they can undergo degradation during manufacturing, storage, and gastrointestinal transit. Furthermore, their practical use is restricted by poor aqueous solubility, low chemical stability, susceptibility to oxidation, and insufficient bioavailability. Accordingly, microencapsulation has become an important strategy to protect labile compounds and to control their release in food, nutraceutical, and pharmaceutical systems [1,2,3].

Natural polymers have been investigated as encapsulation carriers due to their biocompatibility, biodegradability, and generally recognized safety (GRAS) properties. Among these materials, alginate has become one of the most widely studied biomacromolecules for the development of delivery systems. Alginate is a naturally occurring linear anionic polysaccharide mainly extracted from brown algae and composed of β-D-mannuronic acid (M) and α-L-guluronic acid (G) residues arranged in MM-, GG-, and MG-block sequences. The molecular structure of alginate, its molecular weight, and the ratio of mannuronic to guluronic acid residues (M/G ratio) influence its physicochemical behavior such as gel strength, swelling characteristics, porosity, and permeability. Alginate can form three-dimensional hydrogel networks through ionic crosslinking with multivalent cations (most commonly Ca^2+^ ions) owing to these structural features. This mild gelation process, as well as the intrinsic properties of alginate, such as non-toxicity, low cost, and biocompatibility, makes alginate a highly attractive macromolecule for encapsulating a wide range of bioactive compounds [1,2,3,4,5].

Ionic gelation is one of the most employed approaches to produce alginate microparticles since it is simple, aqueous based, relatively inexpensive, and compatible with thermolabile and chemically labile actives. Depending on formulation and processing conditions, ionic gelation can yield beads, microspheres, microgels, and related particulate matrices with different morphologies, internal architectures, and barrier properties. Encapsulation performance in this kind of system is not affected by process variables alone. There is a complex interaction between alginate molecular structure, crosslinking chemistry, particle formation conditions, and the physicochemical nature of the entrapped bioactive compound. Therefore, a comprehension of the structure–processing–property relationship is essential for developing alginate-based delivery systems.

This review adopts an integrated structure-gelation-function perspective unlike previous reviews that primarily focus on alginate hydrogels, microparticle fabrication methods or encapsulation performance separately. Emphasis is placed on linking alginate molecular characteristics (M/G ratio, molecular weight and block distribution) with coordination-driven ionic gelation mechanisms, junction-zone formation, network architecture, mass-transfer behaviour and ultimately bioactive delivery performance. This review provides a mechanistic framework for the rational design of alginate-based delivery systems by connecting molecular-scale interactions to microscale network structure and macroscopic release characteristics.

## 2. Structure and Molecular Characteristics of Alginate

### 2.1. M/G Ratio

**Figure 1:**
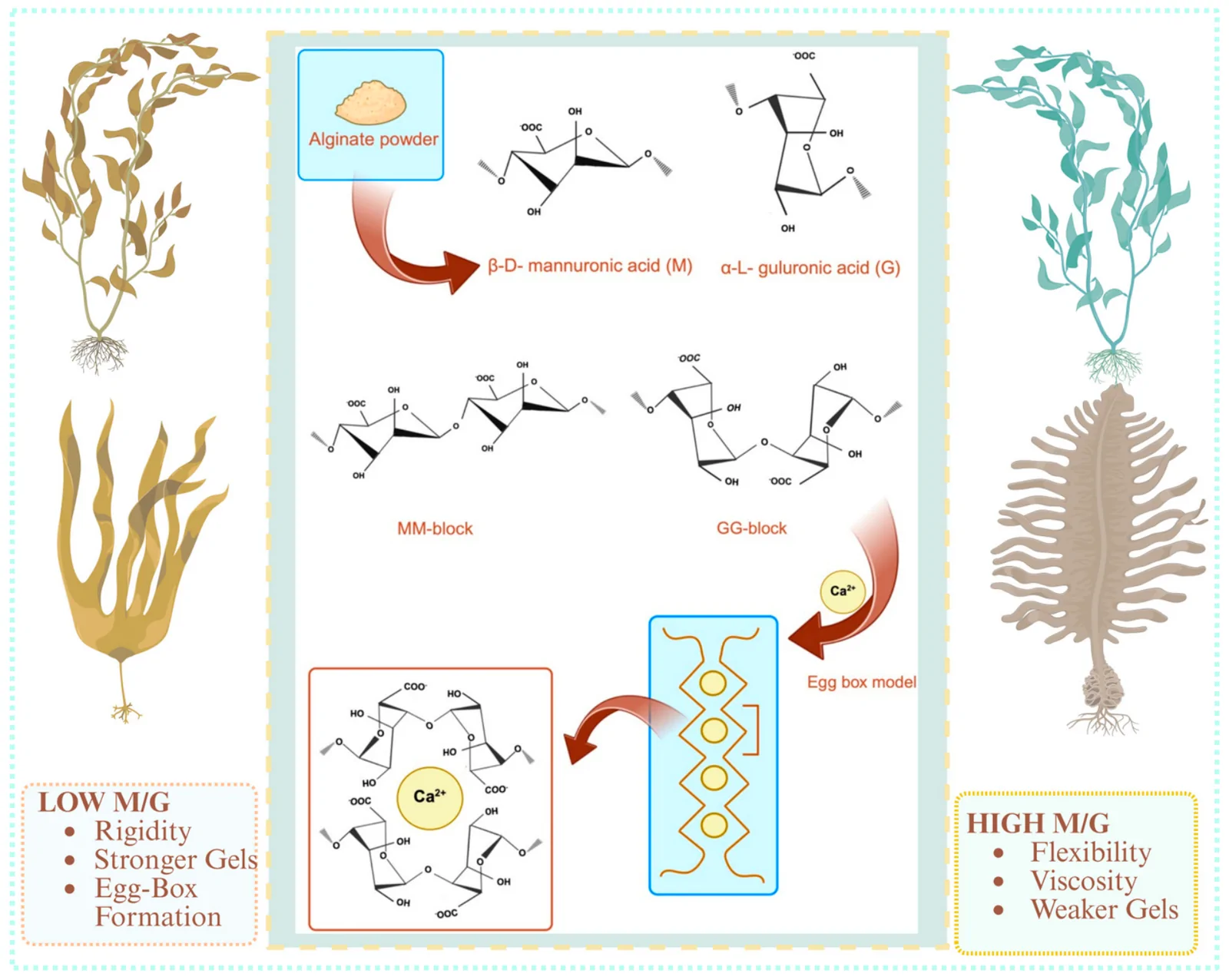
Schematic representation of the molecular structure of alginate, including β-D-mannuronic acid (M) and α-L-guluronic acid (G) residues, MM- and GG-block arrangements, and the calcium-mediated egg-box crosslinking model. Adapted from Kenny et al. (2025) [24] under the Creative Commons CC BY license. Created in BioRender. Bozkurt, R. (2026). https://BioRender.com/v3bp2am.

Alginate, a natural polymer extracted from algae, is considered a promising material because of its natural abundance. Made up of (1,4)-linked β-D-mannuronic (M) and α-L-guluronic acids (G), alginates can create diverse structures with varying molecular weights and physicochemical characteristics. These structures are influenced by the presence of homogeneous blocks (MM or GG) and heterogeneous blocks (MG or GM) [6]. The M/G ratio in alginate’s molecular structure is a key factor influencing its flexibility, gelation speed, and mechanical strength. Alginates rich in G-blocks create firmer, more stable gels due to their stronger attraction to divalent ions, whereas those with more M-blocks produce more flexible networks. Consequently, alginates with higher G-block content are more likely to form stable hydrogels [7,8]. The specific interactions between G residues and metal ions form the basis of alginate’s gelling ability. This process is explained by the “egg-box” model, in which divalent cations, such as Ca^2+^, form coordination bonds with the carboxylate and hydroxyl groups of neighboring G-blocks in the polymer chains (Figure 1). In this model, the G-blocks form ionic crosslinking regions, creating a regular and stable hydrogel network [9,10,11]. The way the G-blocks are structured places the carboxyl groups in an optimal position for metal ion binding, which boosts the gel’s mechanical strength. Additionally, this crosslinking involves not just ion–polymer interactions but also intra- and intermolecular hydrogen bonds [12,13]. The affinity of cations for alginate depends on factors like their radius, valence, and electronic structure. For instance, Ba^2+^ shows stronger binding than Sr^2+^ and Ca^2+^, which greatly influences the mechanical stability of the hydrogels formed. These multivalent cations connect with negatively charged carboxylate groups, creating physical crosslinks between polymer chains [7,14].

In addition to these molecular interactions, the performance of alginate varies significantly depending on the M/G ratio. Alginates with a high M/G ratio can accelerate drug release by forming looser gel networks [15]. Consequently, the M/G ratio emerges as a critical parameter in adjusting both mechanical strength and release profiles in alginate-based delivery systems [16]. Additionally, this ratio could affect the biocompatibility of alginate, since alginates with high M content have been reported to be more immunogenic and substantially more effective at inducing cytokine production than those with high G content [17]. In this context, careful evaluation of the M/G ratio when selecting the appropriate alginate for a specific application plays a significant role in achieving the targeted mechanical properties and controlled release behavior.

Although the M/G ratio and block distribution vary naturally with algal species, geographical origin, and processing conditions, the structure and functionality of alginate can be further tailored through post-extraction modification strategies [18]. Importantly, direct control of the M/G composition should be distinguished from conventional chemical functionalization. Enzymatic epimerization provides a direct route for controlling alginate microstructure through the conversion of β-D-mannuronic acid (M) residues into α-L-guluronic acid (G) residues, thereby modifying the relative distribution of M and G residues along the polymer chain [19]. For example, Mørch et al. demonstrated that C-5 epimerase-mediated conversion of M residues into G residues substantially increased the G content of alginate and altered its block distribution, thereby enabling systematic modulation of the mechanical properties of the resulting gels. In particular, AlgE1-mediated epimerization of mannuronan produced an alginate containing 80% G and resulted in the highest Young’s modulus among the epimerized mannuronan samples examined [20]. In contrast, conventional chemical modifications primarily target the hydroxyl and carboxyl groups of the alginate backbone rather than directly converting M residues into G residues. Representative strategies include oxidation, esterification, sulfation, amidation, phosphorylation, and grafting, which enable the physicochemical and functional properties of alginate to be tailored for different applications [21]. For example, periodate oxidation introduces aldehyde functionalities through cleavage of vicinal hydroxyl groups and can alter molecular weight, chain flexibility, and degradation behavior, whereas esterification and amidation can modify hydrophobicity, mechanical properties, molecular interactions, and self-assembly behavior [21,22]. Sulfation represents another relevant strategy, in which sulfate groups are introduced onto hydroxyl residues, inducing conformational changes and imparting additional biological functionalities [21]. Notably, chemical functionalization may also be associated with changes in the experimentally determined M/G ratio. Cong et al. reported that sulfation of alginate isolated from *Sargassum fusiforme* was associated with an increase in the experimentally determined M/G ratio from 9.0:1.0 to 17.7:1.0, with structural analysis indicating preferential sulfate substitution at the C-3 position of M residues [23]. Collectively, these findings demonstrate that enzymatic regulation of M/G composition and chemical functionalization of the alginate backbone constitute distinct but complementary approaches for tuning alginate structure–property relationships. Such tailoring is particularly relevant to drug delivery and biomedical hydrogel systems, in which gelation behavior, mechanical integrity, degradation, stability, and molecular transport must be balanced according to the intended application [19,21].

### 2.2. Molecular Weight

The molecular weight of alginate substantially affects its rheology. Gels with higher molecular weights have a higher elastic modulus. High-molecular-weight polymer chains form stronger gel networks [25,26]. It is stated that the molecular weights of alginates, with a degree of polymerization from 50 to 3000, range approximately from 10 to 600 kDa [27]. The molecular weight of alginate depends on the extraction process, and higher molecular weights can improve the gel’s physical characteristics [28]. Typically, commercial sodium alginate has a molecular weight ranging from 32,000 to 400,000 g/mol, with high-molecular-weight alginates showing greater shear viscosity [29]. This situation directly affects the physicochemical properties of alginate, such as flow behavior, gel formation, and water-holding capacity. In particular, high-molecular-weight alginates exhibit higher viscosity, which can lead to shear-thinning behavior [30,31]. Conversely, the role of low-molecular-weight alginates in viscosity is rather little, while high-molecular-weight alginates create a denser and more stable network structure through enhanced chain entanglement [32]. Therefore, the molecular weight greatly influences both the rheological behavior and the emulsifying properties of alginate [33]. The molecular weight of alginate influences both its mechanical properties and cellular behavior, which are essential for biomedical uses [34].

### 2.3. Physicochemical Properties

Physicochemical properties of alginate are mainly controlled by structural parameters, such as molecular weight, M/G ratio, degree of acetylation, and block distribution. These factors collectively influence critical performance characteristics, such as rheological behavior and gel formation [35]. Especially, viscosity and flow behavior are important for the processability and application performance of alginate-based systems. Alginate solutions are generally known to exhibit non-Newtonian shear-thinning (pseudoplastic) behavior, which is mainly attributed to the difference in molecular weight and chain length distribution [36]. In addition to these structural features, the chemical composition and the affinity of alginate for divalent ions strongly influence its physicochemical behavior. Alginates are polysaccharides that are generally soluble in water, but their solubility is very much influenced by environmental conditions, such as pH and the concentration of divalent ions in the medium. For example, at pH values lower than 3.5, intramolecular hydrogen bonds are more likely to form, resulting in gelation. On the contrary, the presence of a high concentration of divalent ions induces ionic crosslinking between polymer chains and, therefore, gelation and a decrease in solubility [8]. The kinetics of alginate gelation also depend on the type of crosslinking agent used. Highly soluble calcium salts, such as CaCl_2_, induce rapid gelation, whereas less soluble sources, like CaCO_3_ or CaSO_4_, enable a slower, more controlled, and homogeneous gel formation process. This distinction is particularly important for applications such as injectable hydrogels and bioprinting, where precise control over gelation is required [37]. Furthermore, crosslinking has a pronounced effect on the mechanical properties of alginate-based materials. Recent studies have demonstrated that calcium-mediated crosslinking significantly enhances the tensile strength of alginate films. This improvement is attributed to the reinforcement of intermolecular interactions between polymer chains, resulting in increased structural integrity. Consequently, films with superior mechanical resistance and reduced water solubility can be obtained [38]. The polydispersity index (PDI) is an important measure that characterizes the breadth and heterogeneity of the molecular weight distribution in a polymer system [39]. A high PDI means a broad molecular weight distribution of the polymer chains, while a low PDI means a narrower and more uniform distribution. PDI is defined as the ratio of the weight-mean molecular weight to the numerical-mean molecular weight and hence is a quantitative measure of the breadth of the molecular weight distribution of the polymer [40]. The determination of the PDI in alginate systems is essential to understand the chain length distribution, which is directly related to the processability, rheological behavior, and mechanical properties of the material [41]. The high molecular weight fractions have a substantial effect on the viscosity and flow behavior of the system, and they dominate the flow behavior, especially in the systems with a wide molecular weight variation [32]. This is consistent with research that has shown that polydisperse systems can have elongation-dominating flow behavior [42]. Thus, the molecular weight distribution of alginate and, therefore, the PDI value, is key in determining physicochemical parameters and specifically viscosity, with a direct impact on the performance of the material, especially in sensitive applications, such as bioprinting. Table S1 summarizes the physicochemical characteristics of alginates obtained from different algal species collected from various geographical regions.

## 3. Alginate Gelation Mechanism

**Figure 2:**
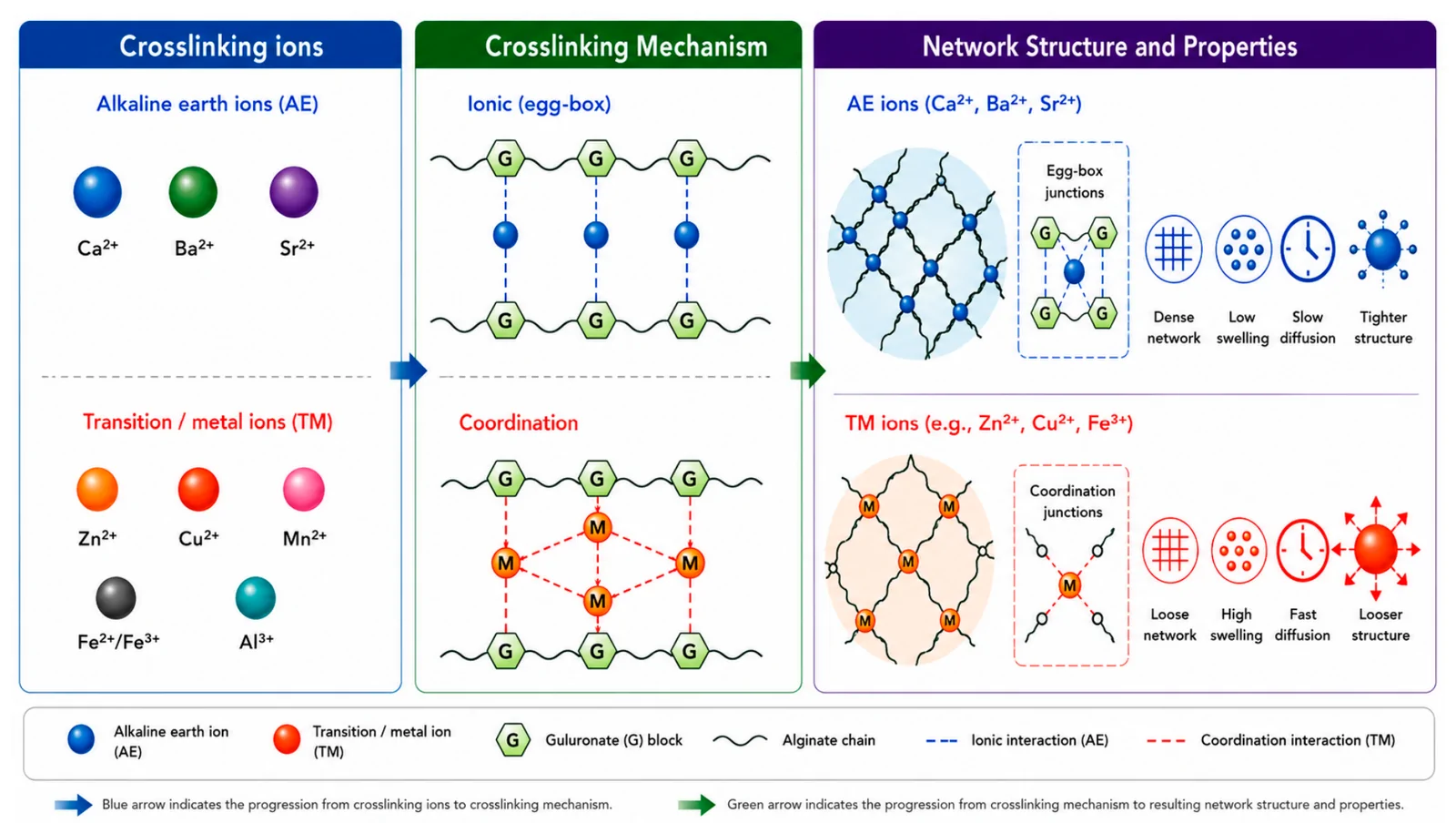
Ion-specific crosslinking mechanisms and resulting network structures in alginate-based systems.

Alginate gels are formed through ion-induced associations of guluronic and mannuronic acid blocks into three-dimensional networks, where multivalent cations bridge carboxylate groups and drive the transition from viscous solutions to elastic hydrogels. Among these cations, Ca^2+^ remains the most widely used crosslinker in food and biomedical applications, but other divalent and trivalent ions such as Ba^2+^, Sr^2+^, Zn^2+^, Cu^2+^, Mn^2+^, Fe^2+^/Fe^3+^, and Al^3+^ can induce gels with markedly different selectivity for G/M blocks, junction zone structures, and mechanical properties. Building on this general picture, the mechanism of alginate gelation can be further clarified by examining Ca^2+^-mediated crosslinking, the egg-box model of junction zones, and the formation of three-dimensional gel networks, as schematically illustrated in Figure 2 [2,11,43].

Figure 2 schematically illustrates how different multivalent cations generate distinct crosslinking arrangements in alginate networks. Alkaline earth ions such as Ca^2+,^ Ba^2+^, and Sr^2+^ mainly promote egg-box-type junction zones, particularly within G-rich alginate sequences, whereas transition metal ions may establish more complex coordination interactions with alginate functional groups. Consequently, ion identity influences junction-zone architecture and the resulting network properties, including network compactness, swelling behavior, heterogeneity, and diffusional transport. These differences are directly relevant to the encapsulation stability and controlled release performance of alginate-based microparticles.

Systematic studies based on elemental analysis and spectroscopy further show that the interactions of alkaline earth and transition metal ions with alginate influence gelation kinetics and junction zone architecture, with direct consequences for network stiffness, heterogeneity, swelling behavior, mesh size, and diffusional pathways [44,45]. These structural differences provide an important basis for tailoring encapsulation efficiency and release profiles in alginate-based microparticles [2,46].

In addition to these polysaccharide-based interactions, it is worth noting that calcium ions are not limited to alginate systems but can also interact with proteins and mixed biopolymer matrices, where they form ionic bridges, reduce electrostatic repulsion, and promote aggregation, leading to more complex and tunable gel structures. This broader perspective highlights that calcium-induced gelation should be considered as a versatile mechanism extending beyond a single polymer system [45].

Accordingly, understanding these structure–mechanism relationships is essential for the rational design of alginate-based delivery systems. Within this framework, these fundamental aspects are examined in detail in the following sections, starting with Ca^2+^-mediated crosslinking as the primary driving mechanism of alginate gel formation.

For clarity, the main ion-specific differences in alginate binding preference, gelation behavior, network and mechanical properties, advantages, limitations and safety considerations, and representative applications are comparatively summarized in Table 1.

### 3.1. Ca^2+^-Mediated Crosslinking

**Figure 3:**
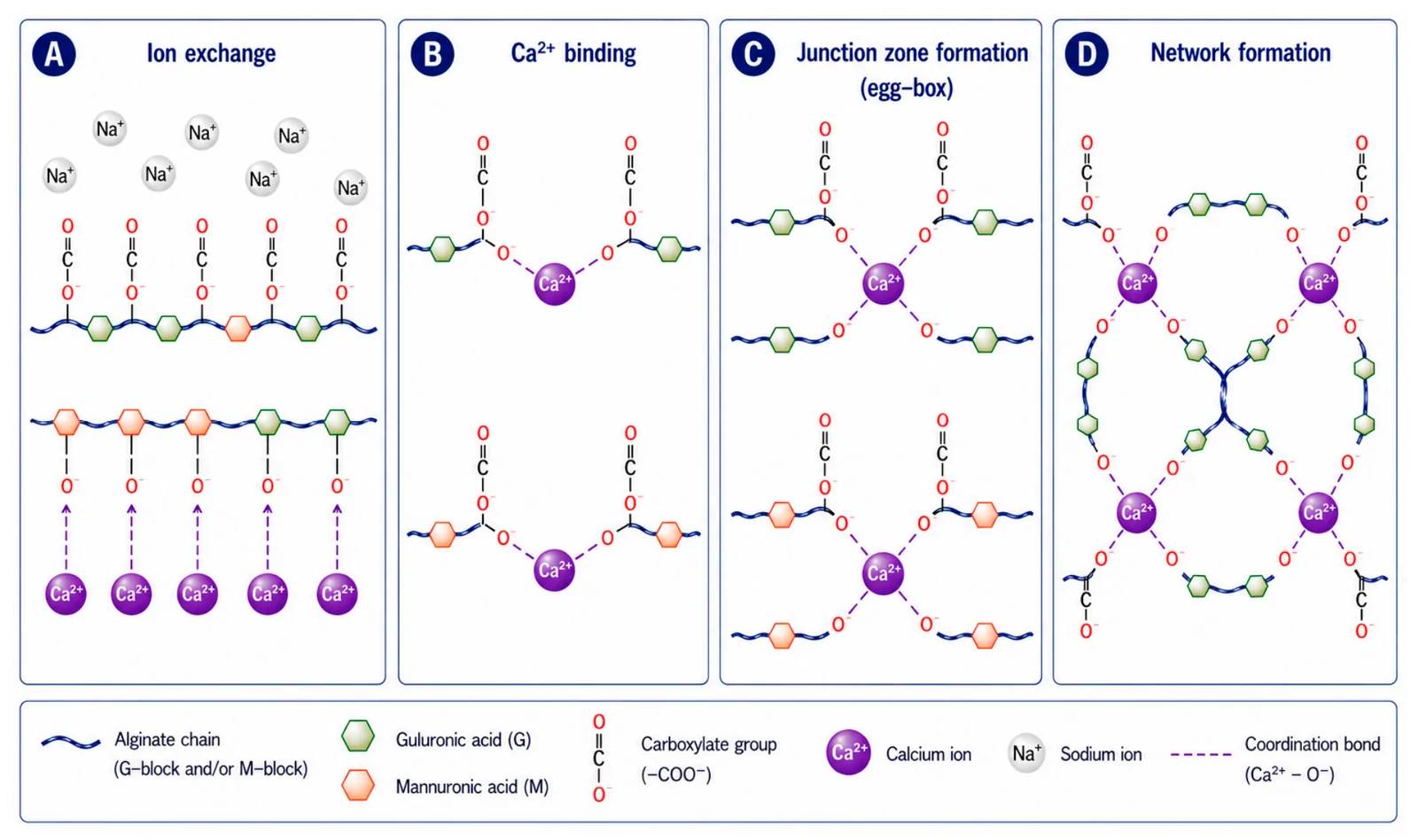
Schematic representation of Ca^2+^-mediated alginate gelation, including (**A**) ion exchange with monovalent counterions, (**B**) Ca^2+^ binding to carboxylate groups and ionic bridge formation, (**C**) nucleation and growth of egg-box-type junction zones, and (**D**) development of a three-dimensional crosslinked network. Arrows indicate the sequential progression of the Ca^2+^-mediated gelation process from ion exchange to three-dimensional network formation.

In Ca^2+^-mediated alginate gels, divalent calcium ions diffuse into the polymer solution and replace the monovalent counterions associated mainly with guluronic acid residues and, to a lesser extent, mannuronic acid residues, forming ionic bridges between neighboring chains [43]. This ion exchange-driven process leads to the formation of junction zones, which form the basic structural units of the gel network. In practice, this is not a single-step process but develops progressively through ion exchange, Ca^2+^ binding, junction zone formation, and network growth, as illustrated in Figure 3.

It is increasingly recognized that this process is strongly governed by the interplay between Ca^2+^ transport and reaction kinetics, rather than being solely controlled by equilibrium binding. As a result, spatial gradients in Ca^2+^ concentration can lead to non-uniform crosslinking, particularly under diffusion-limited conditions [47,50,51].

The rate and homogeneity of gelation depend strongly on Ca^2+^ concentration, the nature of the accompanying anions (e.g., chloride versus lactate), and whether calcium is supplied by rapid external diffusion or by slow in situ release during internal gelation [47,50,51]. Rapid exposure to concentrated CaCl_2_ typically generates steep crosslinking fronts and heterogeneous, shell-like networks, whereas gradual Ca^2+^ release promotes more uniform junction zone formation and mechanically stronger, less brittle gels [47]. This difference in crosslinking pathways is particularly critical for applications requiring precise control over internal structure, as heterogeneous gelation often results in core–shell architectures that directly influence mass transfer and release behavior. Under external gelation conditions, this process is typically diffusion-controlled, with Ca^2+^ transport through the progressively forming gel layer becoming the rate-limiting step.

Beyond this classical ion exchange description, recent studies indicate that Ca^2+^-mediated gelation proceeds through a sequence of temporally distinct stages, including initial ion binding, formation of small junction nuclei, and subsequent growth and rearrangement of these nuclei into extended aggregates. Kinetic analyses based on in situ rheology and scattering techniques show that the characteristic timescales of these stages are highly sensitive to Ca^2+^ activity, alginate concentration, and temperature. Incomplete development of the later rearrangement stage may, therefore, result in mechanically weak and structurally heterogeneous gels [52]. These findings highlight that junction zone formation should be considered as a dynamic and evolving process, where continuous rearrangement at the molecular level contributes to the final mechanical properties of the gel network. At the molecular level, this dynamic behavior is further modulated by the sequence distribution of guluronic and mannuronic units along the alginate chain.

Beyond ion concentration and delivery route, the mannuronic-to-guluronic acid ratio (M/G) of the alginate backbone is a primary determinant of how Ca^2+^-mediated crosslinking proceeds. Alginates with a lower M/G ratio (G-rich) form more continuous egg-box junction zones and thus stiffer, less swellable gels, whereas samples with higher M/G ratios yield softer networks with larger mesh sizes and enhanced chain mobility [38,53].

In addition to CaCl_2_, alternative calcium sources and delivery strategies have been developed to gain finer control over the rate and spatial pattern of crosslinking. Systems based on sparingly soluble salts, such as CaCO_3_, combined with slowly hydrolysing acid precursors, or Ca–chelate complexes that gradually release free Ca^2+^, enable quasi-uniform gelation throughout the volume and mitigate the formation of dense outer shells that are typical for diffusion-controlled external gelation [48,54]. These approaches are particularly attractive for microparticle production, where homogeneous crosslinking is crucial to obtain mechanically robust beads with reproducible size, internal porosity, and release behavior. In such systems, controlling the Ca^2+^ release profile provides an effective strategy to tune not only network homogeneity but also pore structure and diffusional pathways within the gel matrix.

It is also increasingly recognized that Ca^2+^ rarely acts alone in real formulations. In typical systems, monovalent ions, such as Li^+^, Na^+^, and K^+^, together with other background electrolytes, may compete with Ca^2+^ for carboxylate binding sites or screen electrostatic interactions, thereby altering the apparent gelation threshold and affecting the stability of junction zones [51]. Recent studies, therefore, treat the M/G ratio and the calcium-to-guluronate molar ratio (R = Ca/G) as coupled design parameters that govern junction zone density, viscoelastic response and, in multiphase systems, the stability and release behavior of alginate-based gels and emulsions [51,53,55].

Taken together, these findings highlight that Ca^2+^-mediated alginate gelation is a highly tunable, multistage process in which ion exchange kinetics, calcium source, delivery mode, and background electrolyte composition can be engineered to design gels with targeted microstructures and functions [48,51,52]. From a process engineering perspective, this behavior can be interpreted as a structure-property-function relationship, where ion exchange and junction zone formation determine network architecture, which in turn governs mechanical strength, porosity, and mass transfer behavior. This mechanistic framework provides a rational basis for linking molecular-scale interactions to macroscopic performance, enabling the design of alginate-based systems with tailored mechanical properties, porosity, and controlled release behavior.

Building on these insights into Ca^2+^-driven crosslinking kinetics and ion-specific effects, the following section focuses on the molecular architecture of junction zones and the classic egg-box model that underpins alginate gel network formation [44,56].

### 3.2. Egg-Box Model and Junction Zone Structure

**Figure 4:**
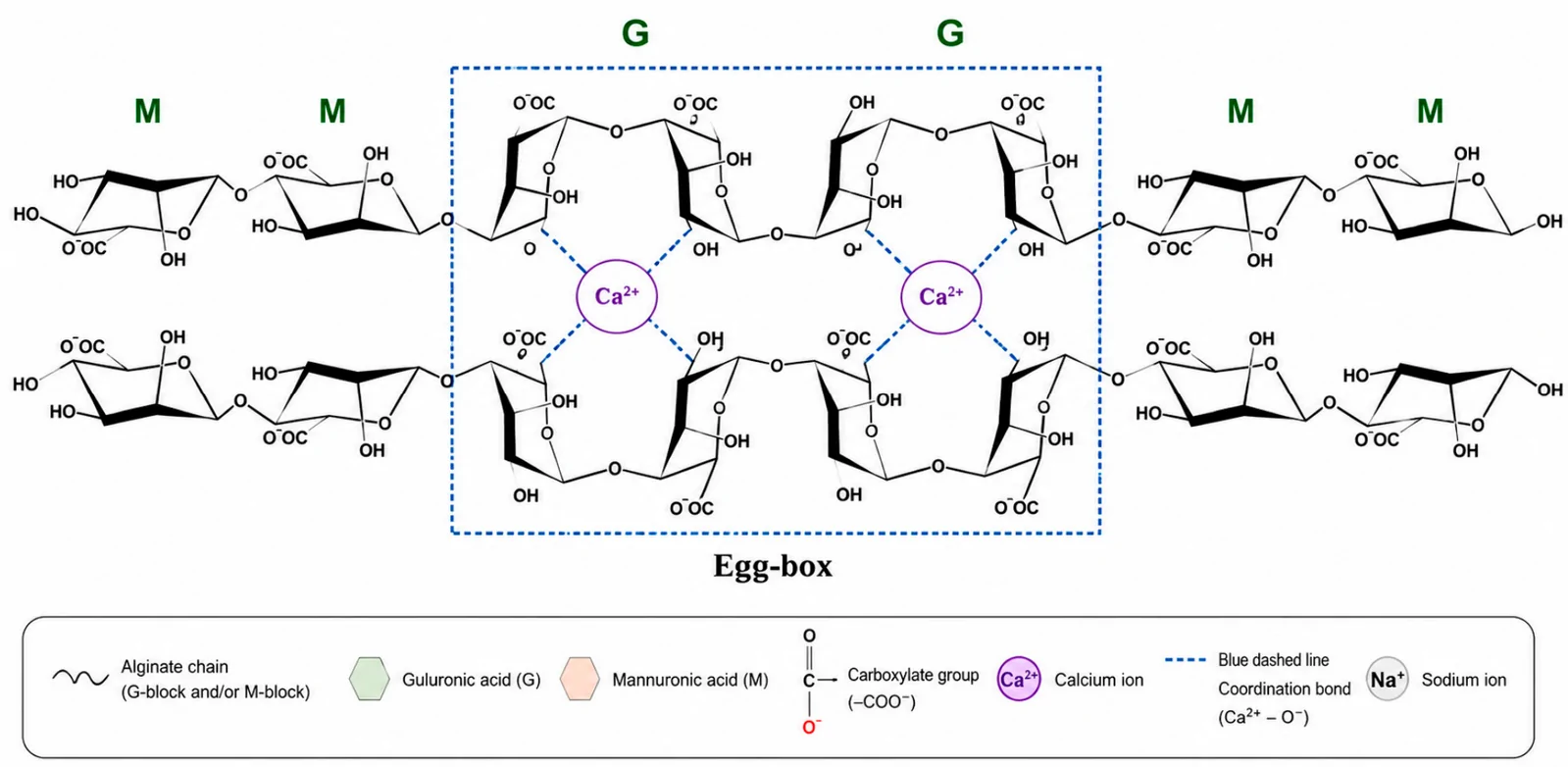
Schematic representation of the egg-box model showing Ca^2+^ coordination between guluronic acid (G) residues of adjacent alginate chains, leading to the formation of junction zones. The blue dashed box highlights the Ca^2+^-mediated egg-box junction region formed between adjacent G-blocks. Adapted from [64] under the Creative Commons Attribution (CC BY 4.0) license.

In the classical egg-box model, neighboring guluronic acid (G) residues on two alginate chains bend into a slightly buckled shape that creates small cavities, and Ca^2+^ ions sit inside these cavities while binding to several carboxylate and hydroxyl groups at the same time [57]. In this way, each Ca^2+^ ion connects multiple G units on and between chains, forming ordered junction zones that hold the network together and explain why gels become stiffer when the alginate is rich in long G-blocks [57,58]. In other words, G-rich segments provide tightly packed egg-box junction zones that act as the main load-bearing elements within the Ca–alginate network [57]. While the classical egg-box model provides a simplified and idealized representation of Ca^2+^-mediated crosslinking, a schematic illustration of this coordination pattern is shown in Figure 4.

Importantly, the classical egg-box model should not be interpreted as a single, static, or universally applicable molecular structure. Rather, it provides an idealized framework for cooperative cation-mediated association between alginate chains, primarily involving aligned G-rich sequences. In practical alginate gels, junction zones can be spatially heterogeneous and dynamically reorganized because their local structure depends on the M/G block distribution, cation identity and concentration, ion-delivery route, pH, ionic strength, and gelation history. Consequently, junction zones may differ in length, cation stoichiometry, chain packing, and connectivity, and can involve short G-blocks and MG sequences in addition to long GG-blocks [44,52,59,60,61,62]. This contemporary interpretation is particularly relevant to externally gelled microparticles, in which diffusion-limited Ca^2+^ transport may produce a highly crosslinked outer region and a less crosslinked inner core [47]. Therefore, the egg-box model is best regarded as a useful structural framework rather than a complete representation of the dynamic and heterogeneous architecture of real alginate networks.

Further structural studies indicate that Ca–alginate junction zones can adopt different helical conformations and packing arrangements depending on gelation conditions, while ion-specific preferences for G-, M-, and MG-blocks contribute to a range of egg-box-like motifs rather than a single unique structure [43,55,63].

Experimental imaging studies have provided further insight into the structural evolution and hierarchical organization of egg-box junctions. Atomic force microscopy (AFM) has been used to investigate Ca^2+^-mediated alginate assembly at the molecular level, revealing a progressive transition from individual alginate chains to dimers and higher-order multimers through monocomplexation, dimerization, and multimerization [61]. Negative-staining electron microscopy has also enabled direct visualization of single alginate chains and their conformational evolution following Ca^2+^ binding, providing microscopic evidence consistent with egg-box-type junction structures [65]. More recently, high-resolution AFM imaging of hydrated alginate hydrogels in aqueous media showed that egg-box dimers assemble into elongated fibrillar structures, which subsequently associate into larger sponge-like networks whose pore characteristics depend on the crosslinking conditions [66]. Collectively, these observations support a hierarchical interpretation of alginate gelation, in which local Ca^2+^-mediated junction formation develops into progressively larger supramolecular network structures rather than remaining as isolated, static egg-box units.

Further work has demonstrated that Ca–alginate junction zones do not necessarily adopt a single idealized conformation but can involve mixtures of 2/1- and 3/1-type helices, as well as contributions from MG segments and short G-blocks, particularly in slowly formed or partially dehydrated gels [58,59]. Complementary diffraction and spectroscopic studies indicate that these mixed helices still satisfy egg-box-compatible coordination geometries but differ in how many G or MG units participate around each cation [58,60]. Elemental analysis-based approaches now allow these different junction motifs to be quantified in terms of cation stoichiometry and local block composition, confirming that junction zones can evolve from simple dimers to extended flat sheets as Ca/G increases [44,67].

Analyses of local composition and ion content indicate that pairs of alginate chains can align side-by-side to form flat sheets of egg-box cavities with different numbers of bound cations, while the relative contribution of GG-, GM-, and MM-based junctions depends on the M/G ratio and metal-ion identity [44,62]. At the molecular level, long GG-blocks preferentially form extended, stiff junction zones, whereas MM and MG regions interrupt or soften these domains and thereby modulate the packing density of egg-box cavities [57,68].

This junction zone architecture is directly reflected in the macroscopic behavior of alginate gels. Networks with many well-ordered egg-box regions, typically obtained from alginates rich in long G-blocks, tend to be stiffer, less swellable, and characterized by smaller mesh sizes, whereas gels formed from alginates with higher M-content and more irregular block distributions are softer, more deformable, and allow faster diffusion of small molecules [44,57]. Recent rheological studies further confirm that increasing the density and continuity of egg-box junction zones shifts the balance from viscous to elastic response and narrows the mesh-size distribution of the network [49,62].

For alginate-based microparticles, this means that by tuning the number, size, and spatial distribution of egg-box junction zones, it is possible to balance mechanical strength with the desired release rate of encapsulated bioactive compounds [44,69].

### 3.3. Gel Network Formation

At the network scale, Ca^2+^-triggered alginate gelation can be viewed as a multistage assembly process in which local egg-box junctions progressively connect into a continuous three-dimensional structure. From a structural perspective, this process reflects a hierarchical organization spanning molecular interactions to macroscopic network formation. Recent work by Haga et al. showed that Ca^2+^-alginate systems pass through three distinct phases, nucleation, intermediate, and gelation, with small fibrillar junction clusters first forming at low Ca^2+^ and then aggregating into thicker fibres at intermediate Ca^2+^ levels and finally developing into a three-dimensional entangled network at higher polymer and calcium concentrations [52]. Circular dichroism-guided phase diagrams revealed that the positions of these transitions depend sensitively on alginate concentration and Ca^2+^ level, with increasing SA concentration (1–6 mg mL^−1^) and Ca^2+^ activity shifting the nucleation-to-intermediate and intermediate-to-gel boundaries to higher Ca^2+^ and ultimately yielding denser, more tightly connected networks. This behavior highlights the strong coupling between ion availability and polymer chain density in determining final network compactness.

At the network level, the mode of Ca^2+^ delivery strongly influences the emerging three-dimensional architecture. Diffusion-controlled external gelation can generate radial gradients with a highly crosslinked outer region and a less crosslinked inner core [70], whereas internal gelation based on the gradual in situ release of Ca^2+^ from systems, such as CaCO_3_-GDL or Ca-EDTA, promotes a more homogeneous crosslink distribution, with fewer radial gradients and improved network uniformity and toughness [48,71]. These differences demonstrate how ion-transport kinetics translate directly into spatial variations in network architecture and mechanical behavior.

In composite systems, such as alginate–gelatin gels, Bäther et al. reported that the sequence and conditions of gelation determine whether alginate-rich and gelatin-rich domains form separate phases or interpenetrating networks, leading to markedly different pore structures and viscoelastic signatures [72]. These findings demonstrate that not only ion dynamics but also polymer–polymer interactions govern the final network architecture.

Quantitative structure–property studies have begun to link these network morphologies to macroscopic mechanical and transport behavior. Scaling analyses and microscopy of Ca^2+^-crosslinked alginate hydrogels show that elastic modulus and failure strength increase with network connectivity and fibril thickness, which are in turn controlled by alginate concentration, M/G ratio, and Ca^2+^ stoichiometry [48,73]. Complementary calorimetric and spectroscopic work indicates that changes in water content and counter-ion type (Na^+^ versus Ca^2+^) affect chain packing and mesh size; more tightly packed networks swell less and slow down the diffusion of small solutes, whereas looser networks exhibit higher swelling and faster release [74]. Across recent reviews on alginate and food hydrogels, a consistent picture emerges: denser, more homogeneous networks provide higher stiffness and erosion resistance, while more open or phase-separated networks offer greater deformability and accelerated transport of encapsulated molecules [71,75,76]. For alginate-based microparticles, these relationships mean that by tuning Ca^2+^ supply mode, polymer characteristics, and the presence of secondary networks, gel network formation can be deliberately steered to achieve the desired balance between mechanical stability, protection of bioactive compounds, and controlled release.

Taken together, alginate gelation conditions establish a direct link between junction zone formation, network architecture, and the resulting functional properties of the gel. Increasing alginate concentration generally increases chain availability and solution viscosity, while the identity and concentration of crosslinking ions determine the extent and organization of ionic junction zones. Crosslinking duration and ion-delivery conditions further regulate ion penetration and spatial crosslinking gradients. These changes influence network density, mesh size, swelling, mechanical integrity, and permeability, which subsequently govern solvent penetration and molecular diffusion through the gel matrix. Thus, variations in formation conditions can ultimately translate into distinct release profiles through their effects on the structure and transport properties of the alginate network.

## 4. Ionic Gelation for Alginate Microparticle Production

Ionic gelation is one of the most widely applied methods for the preparation of alginate-based microparticles because it enables mild, aqueous crosslinking with multivalent cations [43,77]. Building on the coordination and network formation mechanisms described in Section 3, different ion-delivery strategies can be employed to control particle formation and internal network architecture.

By controlling where and how these ions are introduced relative to the alginate phase, several process routes have been developed, including external gelation (extrusion), internal gelation, emulsification-based gelation, and microfluidic production [2,5,78]. Each of these methods offers a different balance between process simplicity, control over particle size and morphology, encapsulation efficiency, and scalability, which determines their suitability for specific applications in drug delivery, food technology, and cell culture [2,5,77].

Overall, the selection of the gelation method is not only a formulation choice but also a process design parameter that directly governs the structural and functional properties of alginate microparticles.

From a process engineering perspective, ionic gelation should be considered not only as a formulation strategy but also as a transport-controlled phenomenon governed by ion diffusion, polymer–ion interactions, and system hydrodynamics.

In this framework, ionic gelation can be further described as a coupled reaction–diffusion process, where the relative rates of Ca^2+^ transport and binding govern the spatial evolution of crosslinking and ultimately determine whether the resulting network develops as a homogeneous or radially heterogeneous structure.

### 4.1. External Gelation

External gelation based on extrusion is one of the most established and commonly used methods for producing alginate microparticles, mainly due to its simplicity, aqueous processing conditions, and compatibility with sensitive bioactives [2,79]. In this approach, a sodium alginate solution containing the encapsulated compound is first shaped into droplets and then exposed to an external gelling bath containing free crosslinking ions, most often CaCl_2_ [43,78].

**Figure 5:**
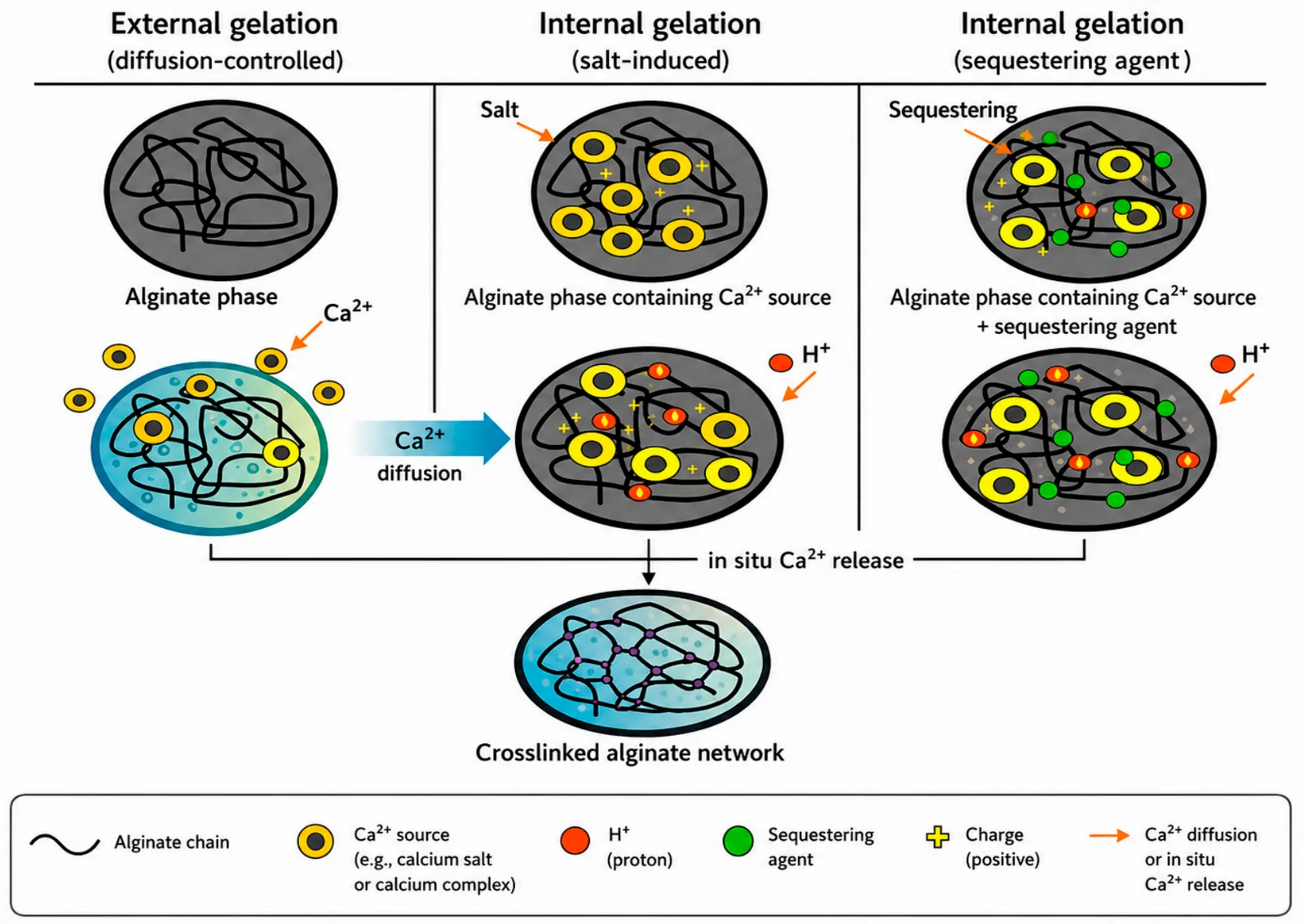
Schematic representation of alginate gelation by external and internal ionic crosslinking. In external gelation (**left**), Ca^2+^ ions diffuse from the surrounding medium into the alginate phase (diffusion-controlled), leading to the formation of a crosslinked shell and, eventually, a Ca–alginate network. In internal gelation (**middle**, salt-induced), an insoluble calcium salt is initially dispersed within the alginate phase and releases Ca^2+^ in situ upon acidification. In internal gelation with a sequestering agent (**right**), calcium salt is combined with a chelating species, and controlled proton release gradually liberates Ca^2+^, resulting in a more homogeneous gel network. The arrows indicate Ca^2+^ diffusion or in situ Ca^2+^ release, while the colored symbols represent the Ca^2+^ source, H^+^, and sequestering agent, as indicated in the schematic.

Calcium ions diffuse from the surrounding solution into the droplets, where they bind to guluronic acid blocks and form junction zones, progressively converting liquid droplets into Ca–alginate hydrogel beads or microspheres [43,44]. This process, as illustrated in Figure 5, involves diffusion of Ca^2+^ ions from the surrounding medium toward the droplet interior, leading to progressive crosslinking from the surface inward. Because diffusion proceeds from the surface inward, externally gelled particles typically exhibit a denser outer shell and a less crosslinked, more hydrated core, which strongly influences their mechanical stability, swelling, and release behavior [43,80]. This inherently diffusion-limited mechanism introduces spatial heterogeneity, which is one of the main limitations of external gelation in achieving uniform network structures.

Such a heterogeneous structure typically results in an initial burst release followed by a diffusion-controlled release phase, which is a key consideration in drug delivery applications. This behavior is particularly critical in pharmaceutical applications, where uncontrolled burst release may compromise therapeutic efficiency and dosage precision.

The simplest configuration of external gelation relies on dripping or extrusion of alginate through a syringe needle or capillary into a gently stirred CaCl_2_ bath, yielding millimeter- to submillimeter-sized beads that are widely used in food, pharmaceutical, and biomedical applications [2,81]. Recent experimental studies have systematically analyzed the influence of alginate properties (molecular weight, M/G ratio) and process parameters such as calcium chloride concentration, flow rate, and needle diameter on bead size and structure, showing that CaCl_2_ concentration and alginate composition are key factors for tuning particle diameter and network density [80,81]. Higher CaCl_2_ concentrations generally promote faster gelation and the formation of smaller, denser beads, although excessive calcium may lead to pronounced shrinkage, surface skin formation and, in extreme cases, erosion or destabilization of the capsules [80,82] In parallel, increasing alginate concentration or using alginates with higher G-content generally enhances gel strength but also increases solution viscosity, which can limit the minimum droplet size attainable by simple dripping methods and may require narrower needles or alternative droplet-generation techniques to reach the low-micrometer range [80]. However, optimizing these parameters requires balancing gelation kinetics with mass transfer limitations, as excessively rapid crosslinking may hinder uniform ion penetration into the droplet core. In conventional dripping or vibrating-nozzle systems, particle diameters typically range from a few hundred micrometers up to several millimeters, and achieving truly monodisperse beads below ~100 µm at high throughput remains technically challenging [77,83]. Furthermore, scale-up of external gelation often relies on multi-nozzle or jet-cutting configurations, which can increase production capacity but also exacerbate issues such as broad size distributions, non-uniform residence times in the CaCl_2_ bath, and heterogeneous crosslinking across batches [77,83].

External gelation by extrusion has also been adapted to more complex matrices and processing environments, extending its applicability beyond simple aqueous CaCl_2_ baths. For instance, extrusion directly into milk or other protein-rich media has been used to produce alginate microcapsules in realistic food systems, where casein and whey proteins, as well as endogenous ions, influence droplet formation, gelation kinetics, and the final mechanical properties of the beads [79]. Other studies have combined external gelation with emulsification, in which alginate is first dispersed as droplets in an immiscible oil phase and then gelled by contacting the emulsion with an aqueous CaCl_2_ solution, enabling the formation of microgel particles with sizes in the low-micrometer range and tailored internal structures [84]. At even smaller length scales, external gelation principles have been integrated into droplet-based microfluidic platforms, where alginate droplets generated in flow-focusing or T-junction geometries are solidified on-chip or off-chip by exposure to Ca^2+^, yielding highly monodisperse microspheres with controlled diameters and narrow size distributions for advanced drug delivery and cell culture applications [85]. Together, these developments illustrate how the classical dripping into CaCl_2_ concept can be refined and combined with emulsification or microfluidics to broaden the accessible particle size range, improve monodispersity, and better tailor the structure–function relationships of externally gelled alginate microparticles [2]. Despite these advances, scale-up of such systems remains a key challenge, particularly in maintaining monodispersity and controlled gelation kinetics under industrial conditions.

### 4.2. Internal Gelation

Internal gelation is an alternative ionic crosslinking route in which calcium ions are generated inside the alginate phase rather than supplied from an external gelling bath, with the aim of obtaining a more homogeneous gel network [43,85,86]. In this method, a sparingly soluble calcium salt, such as CaCO_3_, or a chelated calcium complex (e.g., Ca-EDTA) is first dispersed within the alginate solution, and gelation is subsequently triggered by an acidifying agent or pH shift that releases Ca^2+^ in situ [87,88]. The gradual liberation of calcium ions throughout the droplet volume promotes more uniform crosslinking, which typically reduces the radial gradients in crosslink density that characterize externally gelled beads and can lead to improved mechanical integrity and more predictable swelling and release profiles [44]. This improved structural homogeneity is a major advantage for designing controlled release systems with reduced variability between particles.

In the classical “salt-induced” internal gelation method, CaCO_3_ particles are mixed with alginate and an acid precursor, such as glucono-δ-lactone (GDL); as GDL slowly hydrolyzes to gluconic acid, the pH decreases, CaCO_3_ dissolves, and Ca^2+^ is released homogeneously within the droplets [86,88]. The rate and extent of Ca^2+^ release, and hence the gelation kinetics, can be tuned by adjusting the CaCO_3_/alginate mass ratio and the GDL/CaCO_3_ molar ratio, which has been used to design alginate gels and microspheres with controlled gelation times, internal pH, and porosity [88,89]. From an engineering standpoint, this approach provides a valuable tool for decoupling gelation kinetics from external mass transfer constraints. A more refined variant employs sequestering or chelating agents, such as EDTA derivatives, in which Ca^2+^ is initially bound in a soluble complex and is only freed upon acidification or ion exchange, allowing tighter temporal control over Ca^2+^ availability and gelation kinetics [87,89]. These internal gelation modes, salt-induced and sequestering agent-mediated, are schematically illustrated in Figure 5, where in situ Ca^2+^ release leads to the formation of a crosslinked alginate network throughout the entire droplet volume [87,88].

Internal gelation is particularly attractive for fabricating pH-responsive alginate microspheres, since the more homogeneous network structure can support large, reversible swelling without premature disintegration. For example, internally crosslinked calcium-alginate microspheres produced via water-in-oil emulsification/internal gelation have shown swelling degrees increasing from about 10% at acidic pH 2.0 to approximately 1200% at alkaline pH 8.5, together with sustained and pH-dependent release of loaded drugs, such as simvastatin lactone and simvastatin hydroxyacid [89]. At the same time, internal gelation introduces additional formulation complexity, because the distribution and particle size of the calcium source, the type and concentration of the acid precursor, and the overall ionic strength all influence gelation kinetics and final microstructure [86,87]. If Ca^2+^ is released too quickly, local over-crosslinking or aggregation of partially gelled droplets may occur, whereas excessively slow ion release can result in incomplete gelation or broad particle-to-particle variability; therefore, internal gelation typically requires careful optimization of composition and processing conditions to fully realize its advantages over external gelation [86,89]. Therefore, precise control over formulation parameters is essential to avoid incomplete gelation or structural defects at the microscale.

### 4.3. Emulsification-Based Gelation

Emulsification-based gelation combines droplet formation by emulsification with ionic crosslinking of alginate to produce macro- and microparticulate gels in the low-micrometer size range (typically <100–200 µm) [2,84]. In a typical water-in-oil (W/O) emulsification/internal gelation process, an aqueous alginate phase containing a dispersed calcium source (e.g., CaCO_3_ or Ca-EDTA) is emulsified into an immiscible oil phase using appropriate surfactants; subsequent addition of an oil-soluble acid or pH-modifying agent triggers in situ Ca^2+^ release and gelation of the alginate droplets [87,89]. Alternatively, in external emulsification–gelation, an oil-in-water (O/W) or O/W/O emulsion is first prepared with alginate in the continuous aqueous phase, and gelation is then induced by slowly introducing CaCl_2_ solution, whose ions diffuse to the droplet interface and crosslink alginate to form emulsion microgel particles [2,84]. This approach significantly expands the accessible particle size range compared to conventional extrusion-based techniques.

Emulsification-based methods offer several advantages for alginate microparticle production. The use of high-shear mixers, rotor–stator devices, or membrane emulsification enables the generation of relatively small droplets with tunable size by adjusting emulsifier type and concentration, oil/water ratio, viscosity ratio, and agitation speed. This provides access to narrow particle size distributions in the 10–100 µm range and large interfacial areas, which in turn facilitate rapid gelation and high loading of lipophilic or amphiphilic actives within the dispersed droplets [2]. Such features make emulsification-based systems particularly attractive for encapsulating poorly water-soluble bioactives. Recent work has systematically compared external and internal O/W/O emulsion–gelation routes, demonstrating that external gelation tends to yield smaller emulsion microgels with rougher surfaces, whereas internal gelation produces larger particles with looser internal structures and faster diffusion of entrapped molecules, thereby offering additional levers to tailor rheology, digestion behavior, and release profiles [84,90].

At the same time, emulsification-based gelation introduces specific challenges. Residual oil and surfactant must often be removed by washing and solvent exchange, which can be labor-intensive and may affect the integrity of very soft or highly swollen microgels [2,89]. High shear during emulsification can also distort or damage shear-sensitive biomolecules or cells, so mild emulsification conditions and biocompatible surfactants are required when using these systems for probiotic or cell encapsulation [91]. These additional processing steps may limit the industrial feasibility of the method compared to simpler gelation approaches.

Despite these limitations, emulsification-based gelation remains a powerful and flexible approach for producing alginate microgels and microcapsules with tunable size, internal structure, and release characteristics, and recent systematic and meta-analytical studies on alginate-based emulsion systems underline its growing relevance for controlling lipid digestibility and bioactive delivery in complex food matrices [2,90].

Overall, while external gelation offers simplicity and scalability, internal gelation provides improved structural uniformity, and emulsification-based techniques enable fine control over particle size and morphology. The selection of the appropriate method, therefore, depends on the targeted application, required particle characteristics, and process constraints.

### 4.4. Microfluidic Production

**Figure 6:**
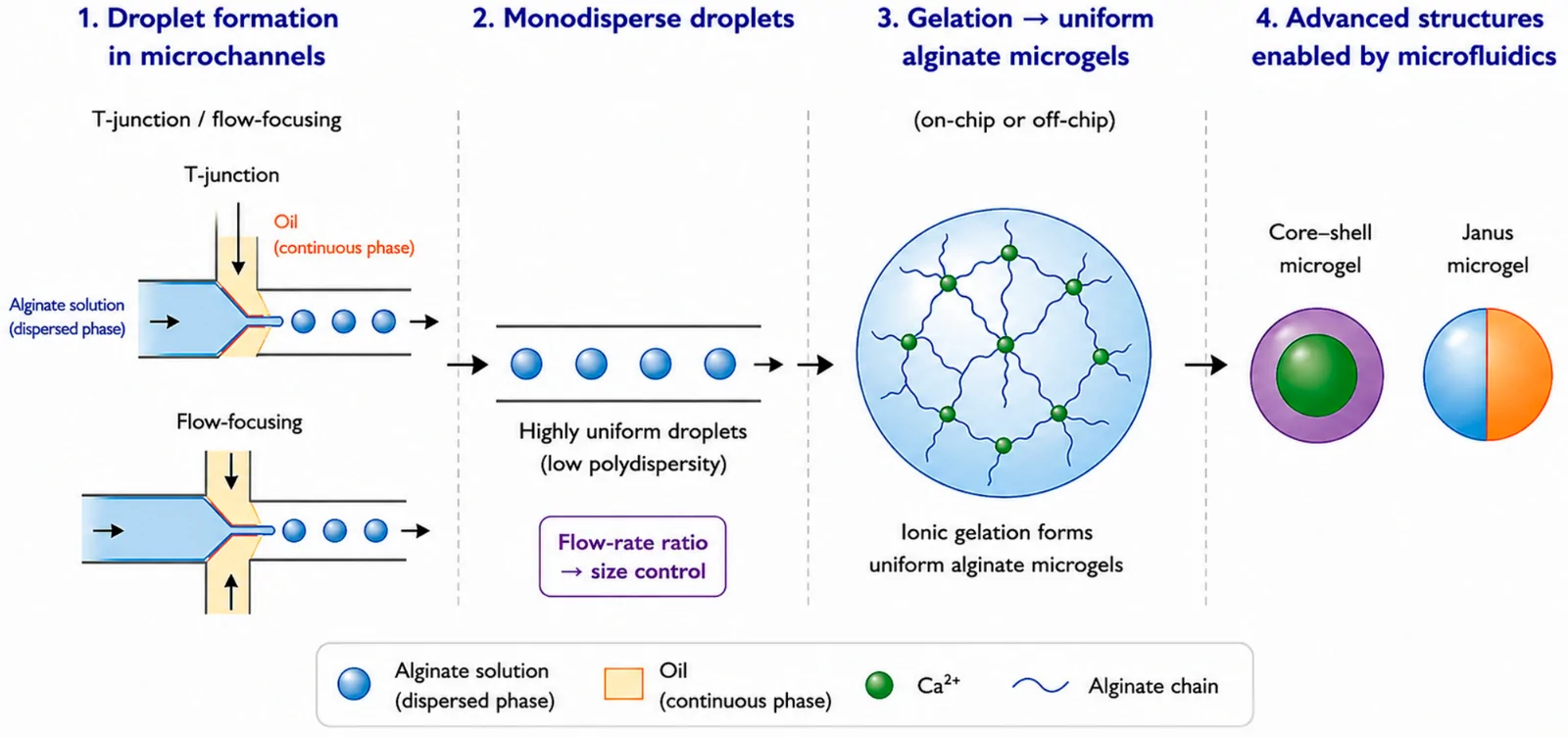
Schematic overview of microfluidic production of alginate microparticles, highlighting controlled droplet generation, size tuning via the flow rate ratio, and the formation of uniform and structured microgels. The arrows indicate the direction of flow, while blue represents the alginate solution (dispersed phase), orange represents the oil (continuous phase), green represents Ca^2+^ ions, and the wavy blue lines represent alginate chains.

Microfluidic production of alginate microparticles relies on controlled droplet formation in microchannels to obtain highly monodisperse microgels with tunable size, shape, and composition [92]. In a typical droplet-based device, an aqueous alginate solution is injected as the dispersed phase and is sheared by an immiscible continuous phase, usually oil, at a T-junction or flow-focusing nozzle [93]. The resulting droplets are then solidified by ionic gelation using either an external CaCl_2_-containing phase or internally released Ca^2+^ [78]. For external gelation, alginate droplets generated on-chip are brought into contact with a CaCl_2_ solution or a CaCl_2_-in-oil emulsion; Ca^2+^ diffuses into the droplets and forms uniform alginate microgels with diameters of about 60–200 µm and coefficients of variation below 3–5%, mainly controlled by the flow rate ratio of the two phases [92,94]. In some microfluidic configurations, even smaller alginate microspheres in the 20–50 µm range with very narrow size distributions have been reported, highlighting the ability of droplet-based microfluidics to achieve precise control over particle size at low polydispersity [78,85]. From a process engineering viewpoint, such devices couple interfacial tension, viscous shear, and residence time in a very controlled way, making it possible to relate operating conditions directly to particle size and monodispersity, which is difficult to achieve with bulk methods [78]. Recent reviews further emphasize that on-chip and off-chip introduction of crosslinkers, as well as internal versus external gelation strategies, allow fine-tuning of gelation kinetics and final microgel morphology within the same microfluidic platform, as schematically illustrated in Figure 6 [78].

More complex channel geometries, such as double or triple emulsions, have been used to fabricate core–shell and Janus alginate microgels with spatially separated compartments [93,95]. These structures enable co-encapsulation of multiple bioactives or separation of core and shell functions; for example, a sensitive probiotic or cell population can be placed in the inner core while the outer shell is tailored to provide mechanical protection and control release [93,96]. Microfluidic routes have also been exploited to generate shape-variable and structurally complex alginate microgels, including biconcave or rod-like particles, by coupling controlled flow conditions with rapid in situ gelation [97,98]. Recent reviews emphasize that microfluidic encapsulation is particularly attractive for probiotics and cell-laden hydrogels because it offers gentle processing, high viability, and precise control over capsule size and shell thickness, as well as over local microenvironments, such as oxygen or nutrient gradients [96,99]. In particular, cell encapsulation studies consistently report high post-encapsulation viabilities and the possibility to engineer pericellular microenvironments by adjusting droplet size, shell composition, and diffusion path lengths [78].

Nevertheless, microfluidic production of alginate microparticles remains largely confined to the laboratory scale due to limited throughput, risk of channel fouling, and the need for specialized equipment and parallelization, which currently restricts its direct use in large-scale manufacturing [78,100]. Droplet-based microfluidic devices typically operate at flow rates of only a few mL h^−1^ per channel, so industrially relevant throughputs require numbering-up strategies that introduce additional complexity and cost [78].

As a result, microfluidic routes are most suitable for high-value applications that require tight control over particle properties, or for generating well-defined model systems to study how alginate microgel structure relates to their functional performance [78,92]. Accordingly, several authors have proposed microfluidic production mainly as a platform for screening structure–function relationships and optimizing formulations, which can then be transferred to more scalable, though less precise, encapsulation technologies [78].

For practical comparison, the main characteristics, advantages, limitations, and application suitability of external gelation, internal gelation, emulsification-based gelation, and microfluidic production are summarized in Table 2.

Overall, method selection should be guided by the required particle size and uniformity, desired crosslinking homogeneity, sensitivity of the encapsulated bioactive, and the intended production scale.

To provide a more quantitative comparison of alginate microparticle systems, representative studies using different ionic gelation approaches are summarized in Table 3. The comparison includes particle size, alginate concentration, crosslinker type and concentration, polymer-to-crosslinker ratio, crosslinking time and temperature, encapsulation efficiency, loading capacity or particle yield, and release characteristics, where reported. These quantitative examples further illustrate the formation–structure–property–release relationship by showing how variations in formulation composition, crosslinking conditions, and processing route are reflected in particle dimensions, encapsulation performance, and subsequent release behavior.

## 5. Factors Affecting Microparticle Characteristics

Alginate-based microparticles are a highly effective platform for the encapsulation and controlled release of bioactive compounds for food, pharmaceutical, and nutraceutical applications. This causes gel networks to form through ionic crosslinking of sodium alginate with divalent calcium ions. Various interactive parameters govern the mechanical integrity and mass transfer properties of these networks, with factors such as the concentration of alginate and its crosslinker and the intrinsic viscosity of the whole alginate solution. These parameters may include particle size, crosslinking density, porosity, and drug release profiles [5,109,110,111]. The literature highlights how modulation of alginate concentration and CaCl_2_ concentration alters the gelation kinetics, while alginate properties also influence processability and microparticle microstructure [3,112,113]. Where discrepancies exist, they often reflect differences in processing mode, oil-based or water-based continuous phases, and the precise measurement of viscosity.

### 5.1. Polymer-Related Parameters

The concentration of alginate directly tunes the solution viscosity and the abundance of guluronic (G) and mannuronic (M) block interactions between two adjacent, conjugated polysaccharides in a crosslinked hydrogel such that both mechanical stiffness and pore structure of microparticles are defined. According to the widely accepted “egg–box” model of alginate gel formation, higher G-content leads to stronger gels with more rigidity because high-affinity binding sites are available for binding divalent cations (especially Ca^2+^). In this respect, alginates with a higher M-content yield less rigid and more elastic structures [17]. Concentrations of alginates with high molecular weights normally raise the viscosity of the solution, which in turn develops larger gelation droplets and, therefore, there will be larger microparticles. Additionally, higher viscosity may improve encapsulation efficiency by reducing diffusion of bioactive compounds into the external phase during gelation [78,114].

Increasing the concentration of alginate directly enhances the viscosity of the solution and promotes the formation of an interconnected polymer network when calcium is used for crosslinking. This results in microparticles that are stronger mechanically, with reduced pore size and altered diffusion pathways, which influence release kinetics [3,113,115].

In practice, studies of this kind (Box–Behnken and others) consistently show that higher alginate levels tend to enhance encapsulation efficiency and modulus, while altering release behavior in predictable ways. This depends on the crosslinking regime and bead morphology [109,110,112]. In inverse-gelation contexts, the concentration and viscosity of alginate together determine residence time and mixing at the oil/water or water/oil interfaces. This, in turn, influences the uniformity of beads and their mechanical stability during gelation [110,116].

Essifi et al. demonstrate that alginate concentration is a primary driver of encapsulation efficiency in calcium alginate microbeads under a Box–Behnken design. They note that higher alginate loading increases entrapment and shaping release potential, while also highlighting the role of gelation kinetics in bead integrity [112]. Toprakçı et al. discuss the optimization of alginate bead formulations for bioactives, emphasizing that alginate concentration strongly influences encapsulation efficiency, and that viscosity, which is controlled by alginate content, modulates diffusion and release [117]. Broader reviews of alginate systems repeatedly cite alginate concentration as a determinant of crosslinking density and gel mechanical properties, which affect release profiles and stability [5,109].

### 5.2. Crosslinking Conditions

One of the most important elements influencing the physicochemical characteristics of alginate-based microparticles made by ionic gelation is the selection of the crosslinking agent. Due to its biocompatibility, low toxicity, and availability, calcium (Ca^2+^) is one of the most commonly used crosslinking agents. Calcium chloride (CaCl_2_) enables rapid gelation under mild conditions, making it particularly suitable for encapsulating sensitive bioactive compounds such as proteins, enzymes, and probiotics [2]. The interaction between Ca^2+^ ions and alginate droplets leads to the formation and inward diffusion of a gel shell, thus producing homogeneous microparticles. Furthermore, this rapid gelation, if not properly controlled, can also result in heterogeneous structures with dense layers and less crosslinked cores [118].

Other divalent cations such as barium (Ba^2+^), strontium (Sr^2+^), and copper (Cu^2+^) aside from calcium have also been studied to alter microparticle properties. Their ionic radius and binding affinity to alginate differ, with a direct influence on the visual impression of gel strength or gel stability [2]. Barium and copper ions have been reported to form stronger gels than calcium due to their higher binding affinity and larger ionic radius. However, the application of these materials is often hampered by toxicity issues, particularly regarding their use for food and biomedical applications.

The amount of crosslinking reagent is also an important determining factor for microparticle properties. When increasing the concentration of CaCl_2_, crosslinking density is increased, producing harder and denser particles. The coupling of polymer chains increases crosslinking density and decreases porosity, which can increase encapsulation efficiency and restrict diffusion of encapsulated compounds during release kinetics. On the other hand, low crosslinker concentrations might result in softer particles that swell more but are less structurally sound [11,113,119].

Beyond the type and concentration of crosslinkers, the long-term stability of Ca^2+^-crosslinked alginate networks must also be considered, due to the thermodynamically metastable nature of Ca^2+^-mediated junction sites. In physiological and food-related environments, non-gel-forming monovalent and weaker-affinity divalent cations (e.g., Na^+^, Mg^2+)^ can displace Ca^2+^ from egg-box junctions via competitive ion exchange. Simultaneously, calcium chelating agents such as phosphate and citrate, naturally oc-curring in gastrointestinal fluids, food matrices, and common buffers, directly disrupt junction integrity by binding free and loosely bound Ca^2+^, since calcium alginate particles are sensitive to chelating agents like phosphate and citrate, and non-gel-forming agents like sodium and magnesium ions. This ion exchange in physiological solution leads to osmotic swelling, resulting in increased pore size, gel destabilization, and rupture [17]. Proteins found in foods or biological environments can also interact with al-ginate chains, and this interaction is highly pH-dependent. At pH values below the protein’s isoelectric point, a positive electrostatic interaction with anionic alginate can occur, while at higher pH values, electrostatic repulsion promotes network expansion and the release of more compounds during digestion [120]. The cumulative effect of these competitive interactions leads to a gradual decrease in crosslinking density, osmotic swelling, pore widening, and ultimately matrix erosion, which directly compromises the mechanical stability and release reproducibility of alginate-based microparticles under gastrointestinal and biomedical conditions. In parallel, it has been reported that unmodified calcium-alginate beads maintain their structural integrity for only a limited time, typically a few hours, during simulated gastrointestinal transit [121]. These stability limitations are among the key factors behind the modified and composite alginate systems discussed in Section 8. These systems utilize secondary coatings such as chitosan or poly-L-lysine and higher-affinity crosslinking ions to increase re-sistance to ion exchange-induced destabilization.

### 5.3. Process Parameters

Core process parameters such as stirring speed, flow rate, nozzle diameter, and the droplet generation method strongly influence particle size, morphology, encapsulation efficiency, and stability. Their effects are closely interconnected and depend on formulation properties such as viscosity, crosslinker concentration, and gelation conditions.

Stirring speed governs shear forces and droplet breakup during emulsification. Higher agitation generally produces smaller and more uniform droplets, whereas excessive shear may cause droplet coalescence, irregular particle morphology, or reduced encapsulation efficiency [122]. In extrusion-based systems, stirring of the crosslinking bath affects ion transport and gelation uniformity; insufficient mixing may lead to aggregation and heterogeneous crosslinking, while excessive agitation can disrupt droplet formation [123,124].

Nozzle or needle diameter is another key determinant of particle size because it defines the initial droplet volume. Smaller diameters generally produce smaller particles, whereas larger diameters yield larger beads under comparable processing conditions. However, the final particle size is also influenced by solution viscosity, flow rate, and gelation kinetics. Therefore, particle formation results from the combined effects of formulation and processing parameters rather than nozzle geometry alone [2,122,125,126].

## 6. Encapsulation of Bioactive Compounds

Alginate microparticles have gained importance as one of the versatile platforms for encapsulation and delivery of a variety of bioactive compounds with different physicochemical properties. Their ability to form hydrogels under mild conditions and tunable structural characteristics, along with biocompatibility, enables them to protect sensitive compounds; their release also could be modulated. Considering the characteristics of encapsulated compounds, including polyphenols, essential oils, vitamins, or pharmaceutical active ingredients, different formulation strategies and system designs are required to accomplish high stability along with efficient encapsulation and release performance. These utilizations of alginate-based delivery systems are associated with food, nutraceutical, pharmaceutical, and biomedical sectors, which underline their multi-functional nature. These application domains are illustrated in Figure S1, while the following subsections describe specific classes of bioactive compounds that can be encapsulated with alginate systems.

### 6.1. Polyphenols

Polyphenols are among the most extensively investigated bioactive compounds in alginate-based delivery systems because of their antioxidant, anti-inflammatory, and antimicrobial properties [127]. However, their susceptibility to oxidation, pH changes, and environmental degradation limits their practical application. Encapsulation within alginate matrices has been shown to improve their stability, protect them during gastrointestinal transit, and enhance bioavailability. Studies involving curcumin, resveratrol, epigallocatechin gallate (EGCG), anthocyanins, and chlorogenic acid consistently demonstrate that alginate-based systems, particularly when combined with chitosan coatings, provide high encapsulation efficiencies and controlled release behavior. These systems, therefore, represent promising platforms for the delivery of polyphenols in functional foods and nutraceutical applications [128,129,130,131,132,133,134,135,136,137].

### 6.2. Essential Oils

The volatility and susceptibility of essential oils to degradation present significant challenges for their industrial application. Alginate-based encapsulation systems offer an effective approach for improving the stability and controlled release of these compounds. Ionic gelation enables the entrapment of essential oil droplets within a crosslinked hydrogel matrix, thereby reducing volatilization and protecting sensitive constituents. Alginate–chitosan systems have demonstrated particularly high encapsulation efficiencies and improved structural stability, highlighting their potential for food, pharmaceutical, and cosmetic applications [138,139,140,141]. Additionally, alginate–chitosan complexes reinforce the capsule structure, yielding enhanced encapsulation efficacy and aiding in more uniform particle formations. As an example, with high encapsulation efficiency (≥90%), pomelo essential oil can be successfully trapped into the alginate–chitosan system [138,142,143]. Black pepper essential oil used for encapsulation showed acceptable encapsulation efficiency, and GC analysis did not detect degradation of terpenes. The results showed that the material offered decent core protection [144].

### 6.3. Vitamins

Many vitamins are highly sensitive to environmental factors, such as light, oxygen, and temperature, which can significantly reduce their stability and bioavailability. Alginate-based encapsulation has been successfully applied to protect vitamins and improve their controlled release characteristics. Studies on riboflavin and vitamin D3 have shown that alginate and alginate–chitosan systems can enhance stability, maintain structural integrity, and provide sustained release profiles. The incorporation of secondary polymers, such as chitosan, further improves mechanical strength and storage stability, making these systems attractive carriers for vitamin delivery [145,146,147].

### 6.4. Pharmaceutical Active Ingredients

Alginate-based delivery systems have attracted considerable interest for pharmaceutical applications because they can protect active ingredients from degradation, improve bioavailability, and enable controlled or targeted release. Encapsulation strategies have been successfully applied to a wide range of therapeutic agents, including poorly soluble drugs, peptide-based therapeutics, and micronutrient formulations. Recent studies indicate that alginate carriers can improve drug stability, regulate release kinetics, and enhance therapeutic efficacy through pH-responsive and diffusion-controlled delivery mechanisms. These properties make alginate-based systems promising platforms for advanced drug delivery applications [148,149,150,151,152,153].

The studies summarized above demonstrate the potential of alginate-based sys-tems to encapsulate various bioactive compounds. However, a balanced assessment requires consideration of significant limitations reported in the literature. Immediate release and insufficient long-term stability are among the recurring problems in these systems. Alginate networks are inherently susceptible to ion-exchange-driven destabi-lization under physiological and storage conditions. This makes precise control of deg-radation and release profiles challenging. Alginate is also a natural polymer whose properties can vary depending on the source and production conditions. Properties such as molecular weight, M/G ratio, viscosity, and purity are significantly influenced by the algal source, harvesting conditions, and extraction protocol. However, many ap-plication-oriented studies do not report these parameters in sufficient detail. This limits the reproducibility of studies and the comparability of results between studies [64]. Furthermore, a significant portion of the evidence summarized in this section has been derived from simulated gastrointestinal or storage conditions. These models may not fully reflect the complexity of the in vivo gastrointestinal environment in terms of en-zymatic, microbial, mechanical, and absorption aspects. Therefore, the conservation or release performance observed in vitro does not always directly translate to in vivo bio-availability [154].

Finally, translational validation is still limited in many of the systems discussed above. Beyond laboratory-scale in vitro studies, there is a need for increased pilot-scale production, animal experiments, and clinical evaluation studies [64]. These limitations should be considered in interpreting the current performance of alginate-based bioac-tive substance delivery systems and in determining future research priorities.

In conclusion, alginate microparticles provide a flexible and biocompatible platform for the encapsulation of diverse bioactive compounds. Through appropriate control of network structure, crosslinking density, and formulation composition, these systems can enhance stability, improve bioavailability, and achieve tailored release profiles for a broad range of applications.

## 7. Release Mechanisms

**Figure 7:**
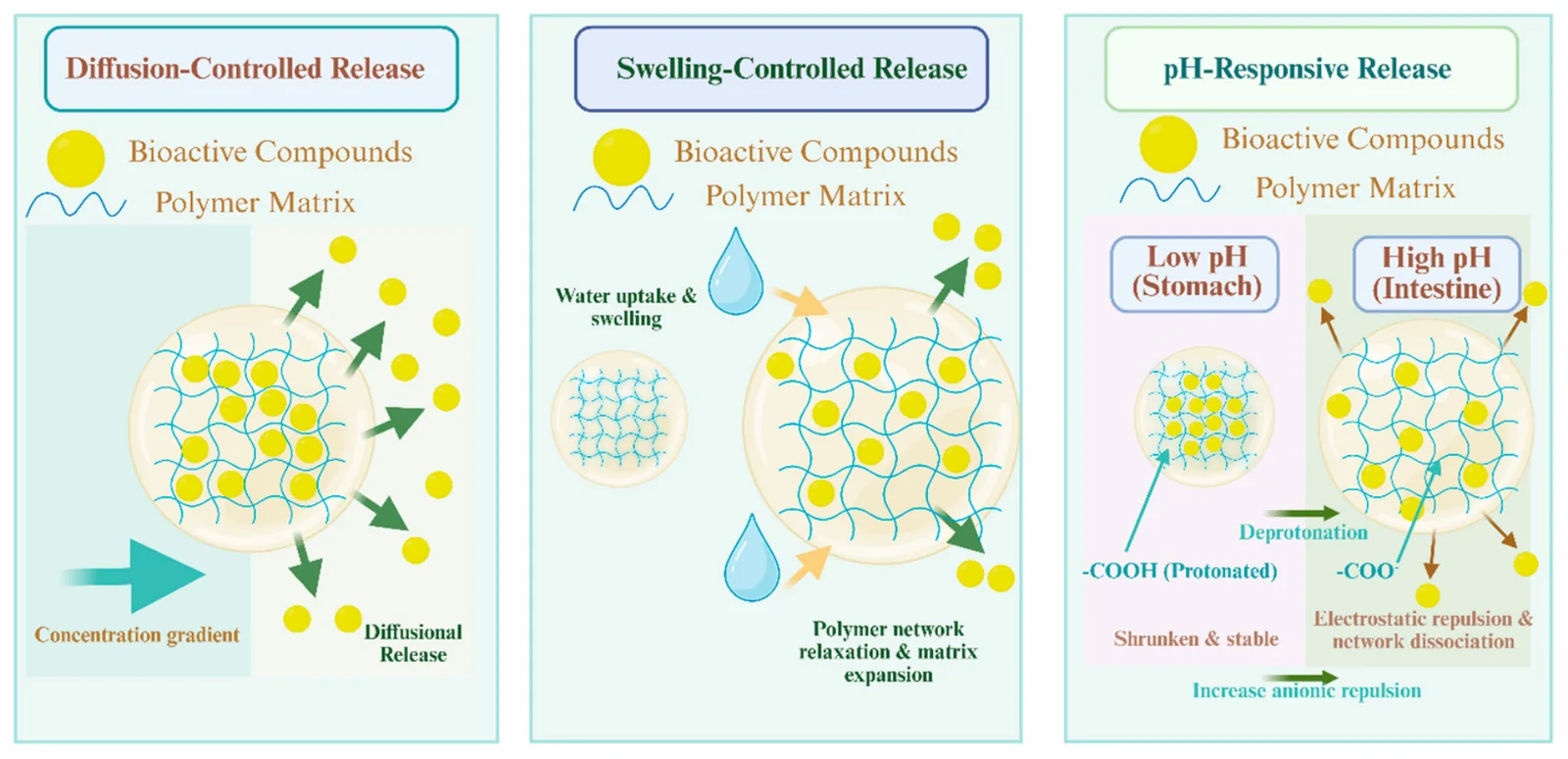
Schematic representation of diffusion-controlled, swelling-controlled, and pH-responsive release mechanisms of bioactive compounds from alginate microparticles. Yellow circles represent bioactive compounds, blue wavy lines represent the polymer matrix, and arrows indicate the direction and underlying processes of bioactive compound release, including diffusion, matrix swelling, and pH-responsive release. Created in BioRender. Bozkurt, R. (2026). https://BioRender.com/18fkvvy.

The release of bioactive compounds from alginate-based microparticles is a multistage process involving the dynamic structure of the polymer network and the complex interaction of environmental factors, rather than a simple leakage [3]. In systems prepared via ionic gelation, the release behavior is directly dependent on the stability of the “egg-box” structure formed by calcium ions with guluronic acid, the internal porosity of the matrix, and the molecular affinity of the trapped bioactive compound with the polymer [155,156]. Current studies emphasize that release is not merely leakage but a structural change resulting from the hydration, swelling, and interaction of polymer chains with ambient ions [157,158]. To accurately predict and tailor delivery performance, it is essential to distinguish among the distinct yet interrelated mechanisms operating within the alginate matrix, including diffusion, swelling and polymer relaxation, matrix erosion, ion exchange-mediated network destabilization, and pH-responsive changes. These mechanisms may occur simultaneously, with their relative contributions depending on the structural characteristics of the alginate network, the properties of the encapsulated compound, and the surrounding environment. The principal mechanisms of diffusion-controlled, swelling-controlled, and pH-responsive release are schematically illustrated in Figure 7.

### 7.1. Diffusion-Controlled Release

Diffusion-controlled release is considered the dominant mechanism for the release of bioactive components, particularly those with low molecular weight and high water solubility, from alginate matrices. This process is described by Fick’s laws, which define the passive transport of molecules along a concentration gradient, and in hydrogel systems, it begins with the penetration of the solvent into the matrix and continues with the diffusion of the bioactive component through the pores in the polymer network [157,159]. While components near the surface or weakly bound to the matrix may initially exhibit a rapid “burst release” profile, hydrogen bonds and electrostatic interactions formed with alginate chains restrict molecular mobility, resulting in a more controlled and sustainable release [157,160]. Furthermore, the diffusion rate is significantly influenced by structural parameters such as pore size, polymer network density, and the degree of ionic crosslinking. Calcium-induced gelation forms a three-dimensional network that restricts molecular mobility, whereas loosely crosslinked structures facilitate faster diffusion. Furthermore, molecular size plays a critical role; smaller molecules diffuse more easily, whilst larger molecules experience steric hindrance within the polymer matrix [3,5,155,157,159].

At the network level, diffusion is more specifically governed by the relationship between the characteristic mesh size (ξ) of the hydrogel and the dimensions of the diffusing molecule. Increasing network density and reducing mesh size enhance steric restriction and can consequently decrease the effective diffusivity (D_eff_) of encapsulated molecules. Accordingly, molecular transport becomes increasingly restricted as the dimensions of the diffusing species approach those of the available network mesh [161,162]. Beyond size exclusion, D_eff_ in alginate hydrogels is also influenced by the characteristics of the available transport pathways, including porosity, constrictivity, and tortuosity, as well as by solute-specific properties and interactions within the matrix [163]. Therefore, diffusion-controlled release should be interpreted as the combined outcome of network architecture and solute characteristics rather than solely as a function of molecular size.

### 7.2. Swelling-Controlled Release

Swelling-controlled release arises from the hydrophilic nature of alginate and its interaction with water. Alginate microparticles exposed to an aqueous environment undergo hydration as water diffuses into the matrix, increasing macromolecular mobility and promoting polymer chain relaxation [157]. Although closely coupled, swelling and polymer relaxation represent distinct contributions to the release process. Swelling refers to the macroscopic dimensional expansion of the matrix resulting from solvent uptake, whereas polymer relaxation reflects the increased mobility and rearrangement of polymer chains during hydration [164,165]. The volumetric expansion of the matrix triggers release by increasing the free volume, which facilitates the diffusion of bioactive components.

The swelling ratio provides a useful macroscopic parameter for relating solvent uptake and network expansion to the structural characteristics of the hydrogel. In calcium–alginate hydrogels, increasing alginate concentration promotes the formation of a denser crosslinked network and has been associated with a marked decrease in the swelling ratio, reflecting restricted solvent penetration and network expansion [162]. Swelling behavior varies depending on parameters such as crosslinking density, polymer composition, ionic strength, and pH [166,167]. In particular, the “egg-box” structure formed by Ca^2+^ ions stabilizes the polymer network, limiting excessive swelling and uncontrolled release. Conversely, high water uptake can sometimes lead to gel erosion, accelerating release kinetics [155,168]. Importantly, erosion-controlled release should be distinguished from swelling-controlled release. Whereas swelling primarily involves hydration and volumetric expansion of the polymer network, erosion involves the progressive loss of matrix integrity and polymer material from the hydrated system. Experimental studies on calcium-induced alginate matrices have demonstrated a close relationship between matrix erosion and the release of incorporated compounds, with increased erosion being associated with faster release [169]. Moreover, the extent of erosion is closely related to network stabilization; stronger interactions between Ca^2+^ ions and alginate can produce a more rigid gel structure, thereby reducing matrix erosion and slowing drug release [170].

### 7.3. pH-Responsive Release

One of the most important advantages of alginate-based systems is the pH-sensitive ionization behavior of the carboxylic acid groups in their structure. In acidic environments (e.g., stomach conditions), protonation of these groups causes the polymer chains to shrink and an insoluble alginic acid layer to form, preventing the premature release of bioactive components [157,171]. Conversely, under neutral or slightly alkaline pH conditions, the electrostatic repulsive forces resulting from the deprotonation of carboxyl groups enable the matrix to swell, the pores to expand, and the bioactive compound to be effectively released, particularly in target regions, such as the intestine [171,172]. Ion exchange-mediated destabilization should, however, be distinguished from this pH-induced swelling mechanism. In Ca^2+^-crosslinked alginate networks, Na^+^ ions present in the surrounding medium can exchange with Ca^2+^ ions associated with the alginate network, resulting in the release of Ca^2+^ from the ionic junctions and progressive weakening of the crosslinked structure. This reduction in ionic crosslinking promotes swelling and destabilization of the alginate matrix and may ultimately contribute to matrix erosion and accelerated release of encapsulated bioactive compounds [173,174]. This pH-responsive behavior enables alginate hydrogels to provide protection in the gastric environment, particularly in oral drug delivery systems, whilst facilitating targeted release in the intestine, and it plays a significant role in enhancing bioavailability [17,175].

### 7.4. Mathematical Models of Release

Mathematical modeling is a critical tool for understanding and optimizing the controlled release kinetics of bioactive components. These models enable the interpretation of experimental data and the assessment of the effects of formulation parameters on release profiles by quantitatively characterizing fundamental release mechanisms such as diffusion, swelling, and erosion. Furthermore, they provide an important framework for predicting in vivo performance from in vitro data [176,177]. In this context, the Higuchi, Korsmeyer–Peppas, and first-order kinetic models are among the most commonly used approaches for analyzing the release of bioactive components from alginate-based systems [157].

#### 7.4.1. Higuchi Model

The Higuchi model assumes that release from polymer matrices is primarily governed by Fickian diffusion and states that the cumulative release amount is proportional to the square root of time. This model successfully describes the early release phases, particularly in solid or semi-solid matrix systems with a constant surface area [178,179]. The simplified form of the Higuchi model is expressed as $Q=k_{H}t^{1/2}$, where $k_{H}$ is the Higuchi dissolution constant, which is related to parameters such as the diffusion coefficient, matrix porosity, and the initial drug concentration [180]. However, the applicability of the Higuchi model is limited to systems where structural changes, such as polymer swelling or matrix erosion, can be neglected, and it is generally valid for the initial ~60% of drug release. Therefore, it may not be sufficient as a standalone model for describing more complex release systems [181].

#### 7.4.2. Korsmeyer–Peppas Model

The Korsmeyer–Peppas model is a semi-empirical approach used to describe release systems in which multiple mechanisms, such as diffusion, polymer relaxation (swelling), and erosion, occur simultaneously. The model expresses the release kinetics using the equation $M_{t}/M_{\infty }=kt^{n}$, where k is the kinetic constant and n is the release exponent that characterizes the underlying release mechanism [182]. The release mechanism can be classified based on the value of the diffusion exponent n: values of n≤0.5 indicate Fickian diffusion, where the release is governed primarily by concentration-driven transport. When 0.5<n<1, the system exhibits anomalous (non-Fickian) transport, reflecting a combined contribution of diffusion and polymer relaxation. An n value close to 1 corresponds to Case-II transport, in which the release is dominated by polymer relaxation processes and often approaches zero-order kinetics. For n>1, super Case-II transport is observed, indicating an accelerated release behavior associated with extensive polymer swelling or structural reorganization. Therefore, this model is widely used for interpreting release mechanisms in systems where swelling and structural changes are significant, such as alginate hydrogels [183].

#### 7.4.3. First-Order Model

The first-order kinetic model assumes that the release rate is directly proportional to the amount of bioactive compound remaining in the system [184]. This model is particularly suitable for describing the release of highly water-soluble compounds from homogeneous matrices. The model is commonly expressed as $ln(C_{t}/C_{0})=-kt$, indicating that the release follows an exponential decline over time. This approach is applicable to systems in which the release rate is not constant but depends on the concentration of the remaining bioactive compound. However, in more complex systems where additional mechanisms, such as swelling or erosion, play a significant role, this model alone may be insufficient, and it is, therefore, recommended to be used in combination with other kinetic models [185,186].

## 8. Modified Alginate Systems

Conventional alginate systems present limitations such as high porosity, relatively weak mechanical strength, and uncontrolled burst release. These resulted from the heterogeneous gel network formed during ionic crosslinking, causing rapid mass transfer and diffusion of the encapsulated active compounds. The high porosity of alginate microbeads results in rapid release, low encapsulation efficiency, and insufficient protection of bioactives under gastrointestinal conditions. Consequently, structural modification of alginate matrices has become essential to enhance barrier properties, increase affinity for hydrophobic drugs, and achieve controlled release behavior [187,188]. So, modified alginate-based systems (hybrid or composite systems) have been developed by combining alginate with complementary polymers and proteins in order to overcome these drawbacks. Therefore, chemical or physical modification of alginate is necessary to improve its functional performance in encapsulation systems [187].

As summarized in Table 4, these modification strategies significantly improve encapsulation efficiency, structural stability, and release performance of alginate-based carriers. The incorporation of secondary polymers or nanomaterials into alginate matrices significantly alters the internal microstructure and transport behavior of the system from a mass transfer perspective [189]. The formation of denser polymer networks and reduced pore size leads to a decrease in effective diffusivity of encapsulated compounds, causing a minimization of premature leakage and burst release [190]. Moreover, the increased tortuosity within the modified gel matrix extends the diffusion path length, contributing to a more sustained and controlled release profile [191]. These structural modifications are particularly important for hydrophobic bioactives, such as curcumin and polyphenols extracted using green solvents, where improved matrix–compound interactions enhance encapsulation efficiency and stability. Compared to native alginate systems, modified alginate-based carriers exhibit significantly improved barrier properties, leading to enhanced protection under gastric conditions and more targeted release in intestinal environments, as summarized in Table 4.

Among the various modification approaches, alginate–chitosan systems are the most extensively studied, as highlighted in Table 4. Chitosan, a cationic polysaccharide, interacts electrostatically with the negatively charged carboxyl groups of alginate, forming a polyelectrolyte complex [205,206]. This interaction leads to the formation of a dense outer layer surrounding the alginate beads, significantly reducing pore size and permeability. In addition to physical and composite-based modifications, chemical modification of alginate has also emerged as an effective strategy. For instance, covalent conjugation of curcumin onto the alginate backbone enables enzyme-responsive release behavior, providing a prodrug-like system with enhanced stability and targeted delivery [204].

To sum up, rational design and modification of alginate matrices enable precise control over mass transfer and release kinetics, effectively overcoming the intrinsic limitations of native alginate systems. Such engineered platforms position alginate-based carriers as highly promising and adaptable delivery systems for next-generation bioactive applications.

## 9. Conclusions

This review focuses on the efficacy of alginate systems, which is fundamentally determined by the interaction of molecular structure, gelation mechanisms, and mass transfer phenomena. Therefore, future research should concentrate more on rational design strategies that integrate material science, process engineering, and application-specific requirements.

The advanced control of mass transfer through precise structural engineering is one of the most promising directions. While conventional ionic gelation produces heterogeneous networks, emerging approaches (like microfluidic-assisted fabrication, 3D printing, and controlled internal gelation systems) enable the production of highly uniform and tunable microstructures. These technologies offer improved control over pore size distribution, crosslinking density and particle geometry, which might facilitate more predictable diffusional transport and release behaviour. Hence, the field is increasingly moving from empirical formulation approaches toward structure-property-function-based design strategies. 

On the other hand, scale-up and industrial implementation remain critical bottlenecks from a process engineering perspective. Although ionic gelation is inherently simple, challenges such as batch-to-batch variability, control of particle size distribution, high solvent-to-feed ratios, and process reproducibility must be addressed. Future studies should incorporate process intensification strategies such as continuous production systems, inline monitoring, and modeling approaches.Computational modelling, data-driven approaches and machine learning have shown potential for formulation optimization and process analysis in biomaterial and hydrogel systems, which might contribute to the future development of alginate-based delivery platforms. 

In addition, standardization and quantitative characterization of mass transfer parameters should be prioritized. Parameters such as effective diffusivity, tortuosity, and permeability are often reported inconsistently, limiting cross-study comparison. The adoption of unified methodologies and modeling frameworks will be essential for advancing the field toward predictive design and industrial translation.

Another important aspect is regulatory and safety considerations. While alginate is generally recognized as safe (GRAS), the incorporation of novel materials (like nanocomposites, DES, and synthetic polymers) requires comprehensive evaluation in terms of toxicity, biodegradability, and long-term stability. Future studies should, therefore, include in vitro/in vivo correlations, bioavailability assessments, and regulatory compliance analyses.

In summary, the future of alginate-based microparticle systems depends on the integration of material innovation, process engineering, and application-oriented design. These systems are anticipated to develop into highly efficient, adjustable, and scalable platforms for the delivery of bioactive substances in the food, pharmaceutical, and biomedical sectors.

## Figures and Tables

**Table 1:** Comparative characteristics of metal ions used for alginate crosslinking.

Crosslinking Ion	Preferred Alginate Interaction/Relative Binding	Gelation, Network, and Mechanical Characteristics	Advantages	Limitations and Safety Considerations	Representative Applications	References
Ca^2+^	Preferential interaction with G-rich sequences; MG-blocks can also participate	Classical egg-box-type gelation; tunable mechanical strength; network density and homogeneity strongly depend on Ca^2+^ concentration, source, and delivery route	Widely available; mild aqueous processing; generally favorable biocompatibility	Rapid external gelation may produce radial heterogeneity and shell–core structures	Food and pharmaceutical encapsulation, cell encapsulation, tissue engineering, and controlled bioactive delivery	[11,43,44,47,48]
Ba^2+^	High affinity for G-rich/GG regions; generally stronger binding than Sr^2+^ and Ca^2+^	Forms highly crosslinked, compact, and mechanically strong networks	High gel strength and network stability	Strong binding may reduce reversibility; safety concerns restrict food and biomedical use	Specialized encapsulation, immobilization, and functional materials	[11,43,44,49]
Sr^2+^	Strong affinity for G-rich/GG regions; generally intermediate between Ba^2+^ and Ca^2+^	Egg-box-type association; forms relatively strong and stable networks	Good mechanical stability; useful where stronger ionic networks are required	Less extensively applied than Ca^2+^; application-specific safety assessment is required	Biomedical and specialized functional materials	[11,43,44,49]
Zn^2+^	Relatively weak affinity compared with Ba^2+^, Sr^2+^, and Ca^2+^; interaction with alginate carboxylate groups	Ion-specific egg-box-type/coordination interactions; generally weaker crosslinking than strongly binding divalent ions	Enables incorporation of Zn-related functionality	Lower binding strength may limit network stability; concentration-dependent safety assessment is required	Functional and biomedical hydrogel systems	[11,43,45,48]
Cu^2+^	Strong alginate affinity; high affinity for G-rich sequences, with ion-specific coordination behavior	Strong crosslinking; can form highly occupied junction zones and mechanically robust networks	Strong crosslinking and potential antimicrobial functionality	Strong binding may alter network transport; concentration-dependent toxicity restricts some applications	Antimicrobial and functional materials, wound-related systems, and environmental applications	[11,43,44,45,48]
Mn^2+^	Relatively weak alginate affinity; interaction is strongly condition-dependent	Forms ion-specific junction structures but generally weaker/less stable networks than strongly binding divalent ions	Potential for specialized functional systems	Low alginate affinity and limited network stability; relatively limited application data	Specialized functional and environmental systems	[11,43,45]
Fe^2+^/Fe^3+^	Coordination with alginate carboxylate groups; Fe^2+^ binding is oxidation- and condition-dependent, whereas Fe^3+^ provides stronger multivalent coordination	Fe^2+^ gelation depends on oxidation state; Fe^3+^ can form strongly crosslinked, mechanically robust networks	Strong crosslinking and potential redox-responsive functionality	Strong dependence on oxidation state, pH, and concentration; application-specific safety considerations	Functional hydrogels, environmental remediation, and stimuli-responsive systems	[11,43,45]
Al^3+^	Strong interaction with alginate carboxylate groups; trivalent-ion binding is less block-selective than typical divalent-ion binding	Strong coordination can produce dense and rigid crosslinked networks	Efficient crosslinking and mechanical reinforcement	Safety/biocompatibility concerns restrict food and biomedical applications	Environmental, adsorption, and specialized functional materials	[11,43,45]

Note: Relative binding strength is qualitative and may vary with alginate block composition, ion concentration, pH, ionic strength, and gelation conditions.

**Table 2:** Comparative characteristics of ionic gelation-based methods for alginate microparticle production.

Parameter	External Gelation	Internal Gelation	Emulsification-Based Gelation	Microfluidic Production	References
Typical particle size	Several hundred µm to several mm	Variable; depends on the droplet generation method	Typically <100–200 µm; commonly 10–100 µm	Typically ~20–200 µm	[2,77,78,80,81,82,85,87,92,94]
Size distribution	Moderate to broad	Moderate; dependent on droplet formation conditions and calcium release kinetics	Relatively narrow; dependent on emulsification conditions	Very narrow; highly monodisperse	[2,77,78,80,83,85,87,92,94]
Crosslinking homogeneity	Low to moderate; radial crosslinking gradients may occur because Ca^2+^ diffuses from the surface towards the particle core	Generally high; gradual in situ Ca^2+^ release promotes more uniform crosslinking	Moderate to high; dependent on whether external or internal gelation is applied	High; droplet formation and gelation conditions can be precisely controlled	[43,44,47,48,78,80,84,86,87,88,89]
Encapsulation efficiency	Bioactive- and formulation-dependent; losses may occur through diffusion into the gelling bath	Bioactive- and formulation-dependent; more homogeneous gelation may improve retention and reduce variability	Bioactive- and formulation-dependent; particularly advantageous for lipophilic or amphiphilic compounds	Bioactive- and formulation-dependent; controlled droplet formation can improve reproducibility	[2,77,80,81,84,87,88,89,90,91,94,96,98]
Throughput	Moderate to high	Moderate	Moderate to high	Low	[2,77,78,92,100]
Scalability	High; multi-nozzle and jet-cutting configurations can increase production capacity	Moderate; additional formulation variables and control of Ca^2+^ release complicate scale-up	Moderate to high; scale-up is possible using high-shear, rotor–stator, or membrane-emulsification systems	Low; parallelization (numbering-up) is generally required	[2,77,78,87,92,100]
Process complexity	Low	Moderate	Moderate to high	High	[2,77,78,84,86,87,88,89,92,100]
Suitability for sensitive bioactive compounds	High; mild and aqueous processing conditions are generally compatible with thermolabile compounds	Generally high; gradual in situ gelation can provide relatively mild and homogeneous processing conditions	Moderate to high; high shear, surfactants, and oil removal steps may limit suitability for shear-sensitive materials, cells, or probiotics	Very high; gentle and precisely controlled processing is suitable for sensitive bioactives, probiotics, and cells	[2,77,84,89,96,99]

Note: Encapsulation efficiency is strongly dependent on the physicochemical properties of the bioactive compound and on formulation and process conditions; therefore, no universal quantitative ranking among production methods is implied.

**Table 3:** Quantitative comparison of representative alginate-based microparticle systems prepared using different ionic gelation approaches.

Preparation Method	Formulation and Crosslinking Conditions	Particle Size	Encapsulated Compound	Encapsulation/Loading/Yield	Release/Performance	Ref.
External ionic gelation	SA/CN/CCT system containing porous starch-loaded gallic acid; SA 1.5–2.5%, CaCl_2_ 1–3%, CN 0.4–0.6%, CCT 0.5–1.5%; CaCl_2_ crosslinking for 30 min	1.5 ± 0.2 mm	Gallic acid	Optimized EE: 56.57%	GA release in SGF: 7.84% for SCC beads vs. 78.49% for SCN beads	[81]
External ionic gelation	SA 1–2% (*w*/*w*); CaCl_2_ 1% (*w*/*w*); 0.45–0.90 mm needle; dropping rate 2 mL/min	1.04–1.44 mm	Sulindac	EE: 88–94%; yield: 87–98%	Complete release within 2 h (1% SA); ~90% release within 4 h (2% SA)	[101]
External ionic gelation	SA 2%; CaCl_2_ 0.2–0.7 M; drug:polymer 1:2–2:1; curing time 3–12 h; gelation at room temperature	0.997–1.436 mm	Mefenamic acid	EE: 79.3–98.99%; yield: 71–89%	10–30% release at pH 1.2 over 8 h; ~100% release at pH 7.4	[102]
Emulsification/internal gelation	SA 2 wt.%; CaCO_3_/SA 10:1 (*w*/*w*); water/oil 1:20 (*v*/*v*); Span 80 5% (*v*/*v*); 1000 rpm	SML: 26 ± 16 µm; SMA: 24 ± 15 µm	Simvastatin lactone (SML); simvastatin β-hydroxyacid (SMA)	EE: 73% (SML); 69% (SMA)	pH-responsive release; relatively slow at pH 2.0 and increased with increasing pH	[89]
Emulsification/internal gelation	SA 2% (*w*/*v*); Ca/alginate 7.3% (*w*/*w*); internal phase 30% (*v*/*v*); Span 80 1% (*v*/*v*); 400 rpm	53.0 ± 12.9 µm	Recombinant human insulin	EE: 75.8 ± 1.8%; loading: 4.18 ± 0.10% (*w*/*w*)	~75% release within 5 min and ~80% at pH 1.2; complete release after transfer to pH 6.8	[103]
Reinforced emulsification/internal gelation	Alginate reinforced with polyanions; representative systems: 0.5% PP or 0.5% DS; chitosan coating also investigated	PP: 68.2 ± 23.3 µm; DS: 71.3 ± 22.9 µm	Insulin	EE: 94.2 ± 2.0% (PP); 101.2 ± 1.5% (DS)	At gastric pH, PP released ~1% insulin in 2 h, whereas DS showed no detectable release; rapid/complete release after transfer to pH 6.8	[104]
Emulsification/internal gelation	SA 2% (*w*/*v*); CaCO_3_-mediated internal gelation; paraffin oil/aqueous phase 50:50; Span 80; 1600 rpm; acetic acid used to release Ca^2+^	4.18 ± 0.28 µm (optimized recovery protocol B3)	Insulin	EE: ~80%; recovery yield: ~70%	Rapid gastric burst: ~100% insulin release within ~5 min at pH 1.2; alginate alone did not provide gastric protection	[105]
Multiple emulsion/ionic gelation	SA 1% (*w*/*v*); Satureja hortensis essential oil 1–3% (*v*/*v*); Tween 80 1% (*w*/*v*); Span 80 1% (*v*/*v*); CaCl_2_ 25% (*w*/*w*); alginate phase prepared at room temperature	73.02–117.65 µm	Satureja hortensis essential oil	EE: 52.40–66.37%; LC: 20.27–26.13%	~60–78% release over ~9 h depending on loading; Fickian diffusion (n = 0.408–0.494)	[106]
Droplet microfluidics/CLEX gelation	SA 1.2%; CaCl_2_/EDTA (30/30 mM) and ZnAc_2_/EDDA (30/30 mM) competitive ligand exchange system; NP:alginate 1:10 (*w*/*w*)	Blank: 125 ± 3 µm; loaded: 106 ± 5 µm	30 nm fluorescent nanoparticles	EE: 70.9 ± 18.9%	55 ± 5% at 30 min; 78 ± 5% at day 1; 92 ± 4.7% at day 2; ~100% at day 4	[107]
Droplet-based microfluidic external gelation	SA 1.5% (*w*/*v*); CaCl_2_ 2% (*w*/*v*); mineral oil + 0.1% Span 80; flow rates 1/15/800 µL min^−1^ (SA/oil/CaCl_2_); final gelation for 30 min at 300 rpm	178 ± 21–375.2 ± 39 µm	BSA	EE: >80%	>50% release within 5 h; 85.8–97.2% release after 72 h	[108]

Abbreviations: SA, sodium alginate; CN, cellulose nanofiber; CCT, carboxymethyl chitosan; SCC, sodium alginate/carboxymethyl chitosan; SCN, sodium alginate/cellulose nanofiber; GA, gallic acid; EE, encapsulation efficiency; LC, loading capacity; SGF, simulated gastric fluid; SML, simvastatin lactone; SMA, simvastatin β-hydroxyacid; PP, polyphosphate; DS, dextran sulfate; CLEX, competitive ligand exchange crosslinking; BSA, bovine serum albumin.

**Table 4:** Studies on alginate-based modified microparticle systems for bioactive delivery.

System	Active Material	Modification Strategy	Key Findings	Mechanism/Effect	References
Chitosan-coated alginate/gellan gum microcapsules	Probiotics	Multi-polymer ionic gelation by vibrational nozzle-assisted extrusion technology	Very high encapsulation efficiency (~97%), improved GI survival and sustained release	Reduced pore size→decreased diffusivity→sustained release	[192]
Soy protein isolate-alginate microcapsules	Probiotics	Water-in-oil emulsion with protein incorporation	Improved mechanical strength and GI stability	Enhanced matrix interactions→delayed diffusion→improved stability	[193]
Chitosan-coated alginate skim milk-coated microspheres	Probiotics	Layer-by-layer coating	High encapsulation (>96%), improved gastric resistance	Dense coating layer→reduced permeability→improved gastric protection	[194]
Alginate/agar and alginate/agar/casein composites	Probiotics	Composite system by ionic gelation	Enhanced viscoelasticity, thermal stability, controlled release	Strengthened network structure→increased tortuosity→controlled diffusion	[195]
Alginate-maltodextrin hydrogel	Probiotics	Composite hydrogel with electrospraying	Improved encapsulation efficiency, stability, storage performance	Matrix reinforcement→reduced molecular mobility→improved stability	[196]
Calcium alginate-pectin beads	Papaya leaf extract	Ionic gelation	High encapsulation efficiency (~87%) and improved morphology	Composite gel network→reduced porosity→enhanced retention	[197]
Alginate–chitosan microparticles	Black locust flower extract	Extrusion with coaxial air flow	High encapsulation efficiency (~92%) and protection of antioxidants	Polyelectrolyte complex formation→reduced diffusion→improved protection	[198]
Alginate-chitosan microparticles	*Viola odorata* Linn. extract	Emulsification + external gelation	High encapsulation efficiency (83.21%) and high stability	Reduced pore size→slower diffusion→enhanced stability	[199]
Alginate–chitosan microparticles	Thyme aqueous extract	Ionic gelation	Increased encapsulation efficiency, improved thermal stability	Matrix densification→reduced molecular diffusion→thermal protection	[158]
Alginate–chitosan microparticles	Orange peel extract	Extrusion with coaxial airflow	High encapsulation efficiency (89%), improved antioxidant delivery	Reduced burst release→controlled diffusion→improved GI delivery	[200]
Alginate–chitosan microparticles	Mangosteen rind extract	Ionic gelation	Very high encapsulation efficiency (~97%), improved colon delivery	Dense matrix formation→delayed release→targeted delivery	[201]
Alginate–chitosan microparticles	Caffeic acid	Microfluidic preparation	High encapsulation efficiency (96.61%), improved thermal stability, and antioxidant properties	Uniform structure→controlled diffusion→enhanced stability	[202]
Alginate hydrogel beads	Curcumin	Emulsion into alginate beads via ionic gelation	High encapsulation efficiency, pH-responsive release	pH-responsive swelling→matrix relaxation→controlled release in intestinal conditions	[203]
Chemically modified alginate (Curcumin-alginate ester)	Curcumin	Covalent esterification	Stable in gastric and intestinal fluids; enzyme-triggered sustained release (~5–6 h) in liver conditions	Esterase-triggered hydrolysis→prodrug→targeted release	[204]

## Data Availability

No new data were created or analyzed in this study.

## Supplementary Materials

The following supporting information can be downloaded at: https://www.mdpi.com/article/10.3390/gels12090774/s1, Table S1: Physicochemical properties of algae obtained from different regions [207,208,209,210,211,212,213,214,215,216,217]; Figure S1: Schematic representation of major application areas and functional advantages in bioactive encapsulation and delivery systems.
