# Supplementary material for: Coordination-Driven Assembly of Alginate Networks: From Egg-Box Structures to Bioactive Delivery Applications
Source: Gels. 2026 Aug 28;12(9):774. doi: 10.3390/gels12090774 (PMC13606149; doi:10.3390/gels12090774)
Supplement: Supplementary file 1 [file gels-12-00774-s001.zip › gels-4504598-supplementary.pdf]

*Review*

# Coordination-Driven Assembly of Alginate Networks: From Egg-Box Structures to Bioactive Delivery Applications

**İrem Toprakçı [1], Ebru Kurtulbaş [1], Rabia Nur Bozkurt [1,2] and Selin Şahin [1,*]**

[1] Department of Chemical Engineering, Faculty of Engineering, Istanbul University-Cerrahpaşa, Avcılar, 34320 Istanbul, Türkiye; irem.toprakciyuksel@iuc.edu.tr (İ.T.); ebru.kurtulbas@iuc.edu.tr (E.K.); rabia.bozkurt@istun.edu.tr (R.N.B.)

[2] Department of Chemical Engineering, Faculty of Engineering and Natural Sciences, Istanbul Health and Technology University, Beyoğlu, 34440 Istanbul, Türkiye

* Correspondence: selins@iuc.edu.tr; Tel.: +90-212-4737070 (ext. 21545)

**Table S1**. Physicochemical properties of algae obtained from different regions.

| Species | Region | M/G Ratio | Molecular Weight ($M_w$) | Alginate Yields (%) | Viscosity | Polydispersity Indices | References |
|---|---|---|---|---|---|---|---|
| *Dictyota mertensii* | North coast of northeastern Brazil | <1 | 278.00±2.66 kDa | 29.28±0.02 | 5.84±0.06 dL/g | - | [207] |
| *Sargassum natans* | Moroccan Atlantic Coast | 1.08 | 0.65x $10^5$(g/mol) | 21.2±0.57% | 1.39 dL/g | - | [208] |
| *Sargassum fluitans* | | 1.27 | 0.37 x $10^5$(g/mol) | 18.1±0.11% | 0.80 dL/g | - | |
| *Cystoseira crinita* | Bulgarian Black Sea | 1.018 | 7.31 × $10^4$ g/mol | 20.18 ± 1.72 | - | 1.38 | [209] |
| *Alaria esculenta* | Bodø | 5.3 ±0.8 | - | 27.3 ±2.2 | - | - | [12] |
| | Tromsø | 3.9 ±0.6 | - | 29.8 ±1.8 | - | - | |

| | | | | | | | |
|---|---|---|---|---|---|---|---|
| | Faroe Islands | 2.4 ±0.5 | - | 26.4 ±5.4 | - | - | |
| | Ireland | 3.9 ±1.2 | - | 43.8 ±5.5 | - | - | |
| *Dictyota dichotoma* | Red Sea Coast of Sudan | 0.92 | - | 8.8±2.12 | 146.96±1.2 (mPa.s) | - | [210] |
| *Hormophysa cuneiformis* | | 0.97 | - | 19.1±1.62 | 109±0.6 (mPa.s) | - | |
| *Padina pavonica* | | 0.93 | - | 7.99±2.60 | 190.73±1.14 (mPa.s) | - | |
| *Turbinaria ornata* | | 1.01 | - | 14.3±1.2 | 140±1.14 (mPa.s) | - | |
| *Padina pavonica* | Djibouti | 1.54 | - | 12.4 | - | - | [211] |
| *Sargassum ilicifolium* | | 1.62 | - | 22.17 | - | - | |
| *Sargassum latifolium* | | 2.07 | - | - | - | - | |
| *Turbinaria decurrens* | | 1.63 | - | 29.3 | - | - | |
| *Punctaria latifolia* | Argentina | 1.09 | 303.6 ± 3 kDa | 17 | 756.7 ± 16 (mL/g) | 1.6 ± 0 | [212] |
| *Nizimuddinia zanardini* | Oman Sea in southern Iran | 1.22 | 119 kDa | 27 ±0.5 | 170.8 mL/g | 2.7 | [213] |
| *Saccharina latissima* | England | 1.98 | 302 kDa | 29.87 ±1.47 | - | 1.367 | [214] |
| *Laminaria digitata* | | 2.23 | 362 kDa | 36.74 ±7.36 | - | 1.223 | |
| *Sacchoriza polyschides* | | 1.83 | 268 kDa | 26.44 ±3.56 | - | 1.659 | |
| *Himanthalia spp.* | | 1.86 | 168 kDa | 32.44 ±0.92 | - | 1.191 | |
| *Laminaria hyperborean(blade)* | Atlantic coast of Morocco | 0.89 | $1.34 \times 10^5$ g/mol | 28.96 ± 0.20 | 2.84 (dL/g) | - | [215] |

| | | | | | | | |
|---|---|---|---|---|---|---|---|
| *Laminaria hyperborean(stipe)* | | 1.38 | 0.66x10$^5$ g/mol | 49.80 ± 2.40 | 1.43 (dL/g) | - | |
| *Saccorhiza polyschides(blade)* | | 1.62 | 0.73x10$^5$ g/mol | 20.19 ± 2.00 | 1.56 (dL/g) | - | |
| *Saccorhiza polyschides(stipe)* | | 2.14 | 0.53x10$^5$ g/mol | 26.28 ± 0.08 | 1.14 (dL/g) | - | |
| *Padina pavonica* | Tunisia | 1.85 | 4890 kDa | - | - | 5.57 | [216] |
| *Saccorhiza polyschides* | Portugal | 0.58 | 6.7 × 10$^4$ kDa | 21 | 33 (mPa·s) | - | [217] |
| *Sargassum muticum* | | 0.49 | 4.9 × 10$^4$ kDa | 23 | 30 (mPa·s) | - | |

References [207–217] cited in Table S1 are listed below.

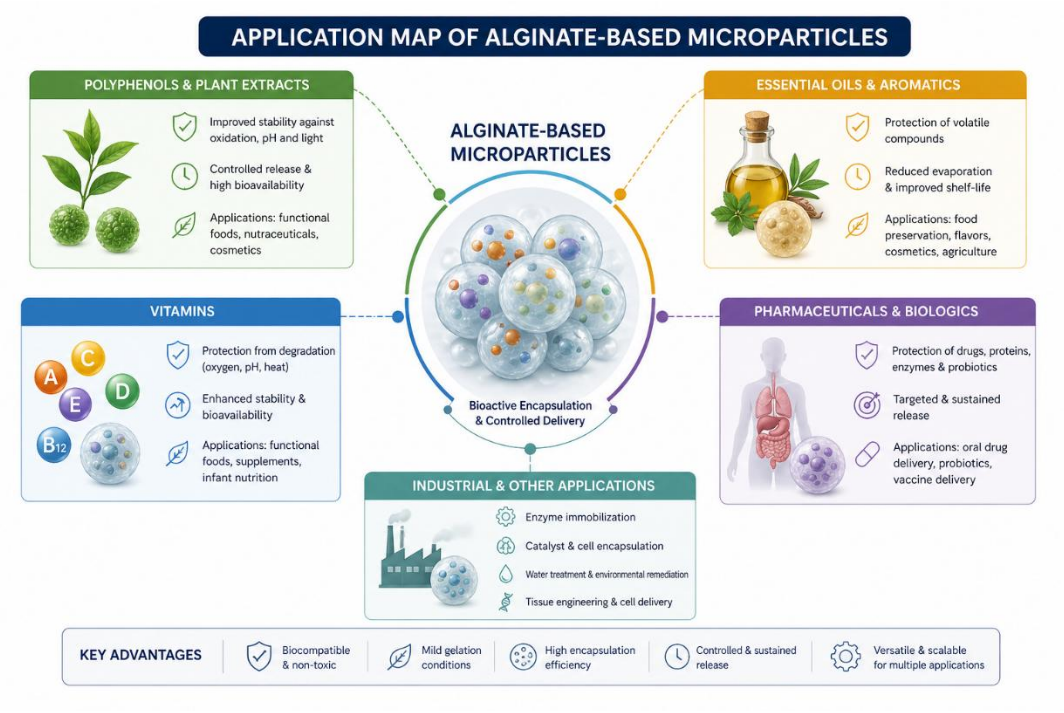


**Figure S1.** Schematic representation of major application areas and functional advantages in bioactive encapsulation and delivery systems.

## References


207. de Oliveira Queiroz, L.P.; Aroucha, E.M.M.; dos Santos, F.K.G.; de Lima Leite, R.H. Optimization of Alginate Extraction Conditions from the Brown Seaweed Dictyota Mertensii Using a Central Composite Design. Algal Res. 2024, 79, 103482, doi:10.1016/j.algal.2024.103482.

208. Kamal, K.; Belattmania, Z.; Khaya, K.; Chaouti, A.; Bentiss, F.; Jama, C.; Stiger-Pouvreau, V.; Sabour, B. Characterization of Alginate from Drifted Pelagic Sargassum Natans and Sargassum Fluitans Along the Moroccan Atlantic Coast. Physchem 2025, 5, 51, doi:10.3390/physchem5040051

209. Kokova, V.; Lukova, P.; Baldzhieva, A.; Katsarov, P.; Delattre, C.; Molinié, R.; Petit, E.; Elboutachfaiti, R.; Murdjeva, M.; Apostolova, E. Extraction, Structural Characterization, and In Vivo Anti-Inflammatory Effect of Alginate from Cystoseira Crinita (Desf.) Borry Harvested in the Bulgarian Black Sea. Mar. Drugs 2023, 21, 245, doi:10.3390/md21040245.

210. Osman, N.; Suliman, T.; Osman, K. Characterization of Native Alginates of Common Alginophytes from the Red Sea Coast of Sudan. Int. J. Second. Metab. 2020, 7, 266–274.

211. Nour, M.; Petek, S.; Daher, A.; Cérantola, S.; Cosquer, N.; Deslandes, E.; Stiger-Pouvreau, V. Bioactive Polysaccharides from Djiboutian Brown Algae: Antioxidant and Anti-Aging Potential for Cosmetic Applications. Cosmetics 2026, 13, 53, doi:10.3390/cosmetics13020053

212. Poza, A.M.; Fernández, C.; Martínez-Gómez, F.; Croce, M.E.; Lezcano, V.; Buschmann, A.H.; Villar, M.A. Punctaria Latifolia as a New Source of Alginate: Structural Characteristics and Biological Activity. J. Appl. Phycol. 2025, 37, 1409–1429, doi:10.1007/s10811-024-03405-9.

213. Abka Khajouei, R.; Keramat, J.; Hamdami, N.; Ursu, A.-V.; Delattre, C.; Gardarin, C.; Lecerf, D.; Desbrières, J.; Djelveh, G.; Michaud, P. Effect of High Voltage Electrode Discharge on the Physicochemical Characteristics of Alginate Extracted from an Iranian Brown Seaweed (Nizimuddinia Zanardini). Algal Res. 2021, 56, 102326, doi:10.1016/j.algal.2021.102326.

214. Silva, M.P.; Badruddin, I.J.; Tonon, T.; Rahatekar, S.; Gomez, L.D. Environmentally Benign Alginate Extraction and Fibres Spinning from Different European Brown Algae Species. Int. J. Biol. Macromol. 2023, 226, 434–442, doi:10.1016/j.ijbiomac.2022.11.306.

215. Kaidi, S.; Bentiss, F.; Jama, C.; Khaya, K.; Belattmania, Z.; Reani, A.; Sabour, B. Isolation and Structural Characterization of Alginates from the Kelp Species Laminaria Ochroleuca and Saccorhiza Polyschides from the Atlantic Coast of Morocco. Colloids Interfaces 2022, 6, 51, doi:10.3390/colloids6040051.

216. Faidi, A.; Becheikh, M.E.H.; Lassoued, M.A.; Stumbé, J.F.; Safta, F.; Sfar, S. Isolation of Sodium Alginate-like Polysaccharide from Padina Pavonica: Optimization, Characterization and Antioxidant Properties. J. Mol. Struct. 2025, 1321, 139737, doi:10.1016/j.molstruc.2024.139737.

217. Mendes, J.C.; Valente, J.F.A.; Sousa, F.; Bernardino, R.; Bernardino, S.; Afonso, C.; Chagas, B. Optimisation of Alginate Ex-traction and Characterisation of Polysaccharides from Brown Seaweed from the Portuguese Coast. Mar. Drugs 2026, 24, 60, doi:10.3390/md24020060.
